# Carbon Monoxide Release from Aryl-Propargyl Dicobalt(0)Hexacarbonyl Derivatives: A Computational and Experimental Study

**DOI:** 10.3390/ijms252111644

**Published:** 2024-10-30

**Authors:** Roberto Paciotti, Cecilia Coletti, Emanuela Berrino, Francesca Arrighi, Alessandro Maccelli, Alba Lasalvia, Maria Elisa Crestoni, Daniela Secci, Simone Carradori, Claudiu T. Supuran, Fabrizio Carta

**Affiliations:** 1 Department of Pharmacy, “G. d’Annunzio” University of Chieti-Pescara, Via dei Vestini 31, 66100 Chieti, Italy; simone.carradori@unich.it; 2 Department of Drug Chemistry and Technologies, Sapienza University of Rome, P.le A. Moro 5, 00185 Rome, Italy; emanuela.berrino@gmail.com (E.B.); francesca.arrighi@uniroma1.it (F.A.); alba.lasalvia@uniroma1.it (A.L.); mariaelisa.crestoni@uniroma1.it (M.E.C.); daniela.secci@uniroma1.it (D.S.); 3 National Centre for the Control and Evaluation of Medicines, Chemical Medicines Unit, Istituto Superiore di Sanità, Viale Regina Elena 299, 00161 Rome, Italy; a.maccelli@gmail.com; 4 NEUROFARBA Department, Sezione di Scienze Farmaceutiche e Nutraceutiche, University of Florence, Sesto Fiorentino, 50019 Florence, Italy; claudiu.supuran@unifi.it (C.T.S.); fabrizio.carta@unifi.it (F.C.)

**Keywords:** CO-RM, cobalt, carbon monoxide release, DFT calculations

## Abstract

In the present study, we focus on dinuclear cobalt-based CO-RMs with the aim of elucidating their CO release mechanism, as well as to understand how structural changes targeted to modify the electronic properties of these compounds can modulate CO delivery. To this end, we specifically synthesized a set of phenyl-propargyl-based CO-RMs bearing –NO_2_, –H, and –OCH_3_ as para-substituents (R) with varying mesomeric influence (M) and different heteroatoms (X = NH, O, or S) linking the propargyl tail and the aromatic ring. The effects of R and X in modulating CO release were assessed by using several experimental and computational techniques to obtain a coherent picture and to shed light on the stability and release properties of Co-based CO-RMs.

## 1. Introduction

**Figure 1:**
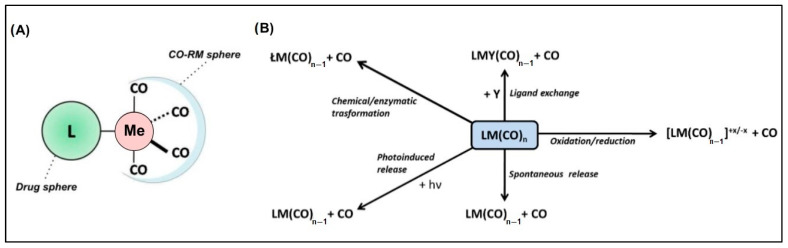
(**A**) General structure of metal-based CO-releasing molecules characterized by the CO-RM sphere (containing the metal atom, Me) and the ligand L, corresponding to the drug sphere region; (**B**) main paths leading to CO release from CO-RMs.

Metal-based carbon monoxide (CO)-releasing molecules (CO-RMs) form a wide class of chemical entities in which a metal atom coordinates several CO groups. These compounds have sparked interest because they potentially represent a system for the controlled delivery of CO in biological environments, with several routes for administration having been explored, like via gas inhalation, with poor results [1,2,3,4,5]. The main biomedical applications of CO-RMs include the management of chronic diseases such as inflammations and cancers [1,2,6,7,8,9,10,11,12,13,14,15], host immunosuppression to circumvent transplant rejection [1,6,7], inhibition of platelet aggregation [1,6,7], vasorelaxation [1,6,7], neurotransmission [13], and anti-bacterial activity [16]. From the structural viewpoint, such molecules consist of a CO-RM sphere, which usually accounts for a Ru, Fe, Mn, Co, or Mo metal species coordinated with Cos, as well as a drug sphere. The latter is an organic scaffold responsible for endowing specific pharmacokinetics, pharmacodynamics, and CO release rates to the entire molecule (Figure 1A) [13,17].

The chemical bond between CO and the metal center is characterized by an overlap between the CO lone pair and an empty metal d orbital (σ donation). A concurrent π backdonation from the occupied metal d orbital to the CO empty π* orbital increases the metal–C bond strength and weakens the C–O bond. Consequently, stronger bonds are formed with metal centers in relatively low oxidation states since they provide filled d orbitals of suitable energy to ease the back bonding [18]. The strength of the metal–(CO) bond in CO-RMs can, therefore, be modulated by the presence of electron-donating or -withdrawing moieties on the drug sphere. A decrease in the metal d electron density results in a weakening of the back-bonding interaction and should facilitate CO release from the metal coordination sphere.

The release of CO may occur in a variety of ways, as schematically depicted in Figure 1B, which are all strictly dependent on the CO-RM structure, the surrounding chemical environment, and possibly a triggering input such as light radiation.

We recently reported and assessed the anti-inflammatory properties of dual-acting Co-containing CO-RMs in-vivo experiments [19,20,21,22,23], providing additional support for the biomedical value of these compounds. Despite extensive efforts, the exact mechanism underpinning the release of CO in Co-based CO-RMs is far from being fully rationalized and commonly accepted by the scientific community [24,25,26].

The main hurdles are the approximation of knowledge of the structural features of the CO-RM moiety and the accurate determination of its chemical nature. In particular, the atomic arrangement of the CoC_2_ in the CO-RM sphere is very close to being unstable and is properly referred to in the Quantum Theory of Atoms in Molecules (QTAIM) as a catastrophic situation [26]. The fragile electronic structure of Co-CO-RMs makes their behaviors highly influenced by the physicochemical environment they are exposed to. This feature heavily differentiates such systems from the naturally occurring and highly stable [Fe-Fe] and [Ni-Fe] clusters in bacterial dehydrogenases [27,28].

Herein, we present a joint computational and experimental investigation focused on the thermodynamics and kinetics of the CO loss process with reference to a small set of simple Co-based aryl-propargyl dicobalt hexacarbonyl (DCH) molecules specifically designed for such purposes.

## 2. Results

### 2.1. Design and Synthesis of CO-RMs

**Figure 2:**
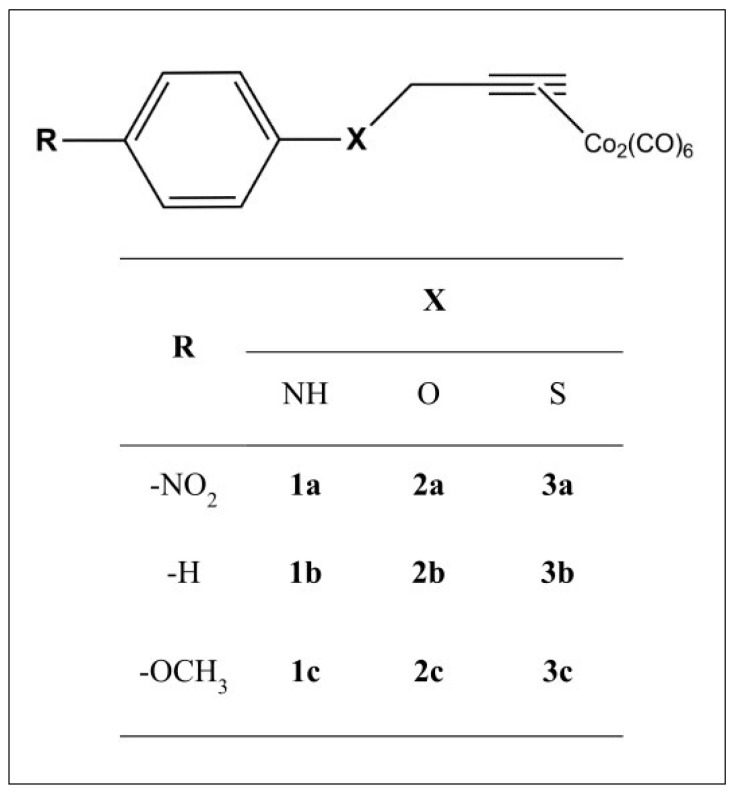
General structure of the compounds considered herein.

We envisaged a chemical template of the type shown in Figure 2. We considered R = –H, as well as -OCH_3_ (an electron-donating group with positive mesomeric effects on the CO-RM sphere (+M)) and –NO_2_ (an electron-withdrawing group with negative mesomeric effects (−M)).

The CO-RM sphere is spaced from the bulky electronic cloud of the aryl ring through a heteroatom (X) bearing unpaired electrons, such as –NH, –O, and –S, making it able to sustain conjugation with the phenyl moiety and to produce electronic effects on the CO-RM sphere.

**Scheme 1:**
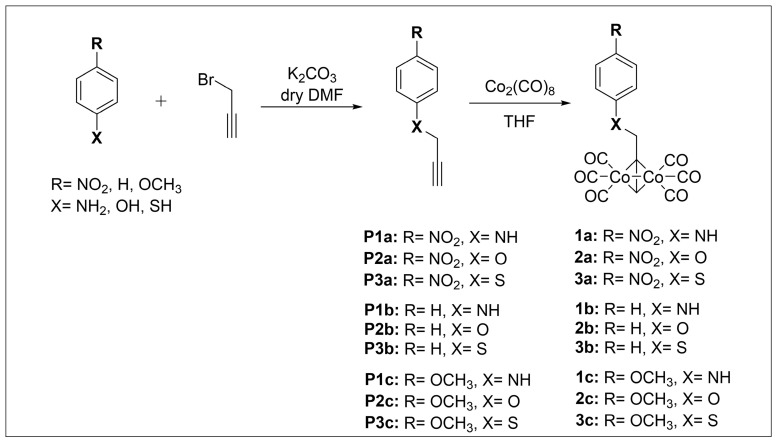
Synthesis of CO-RMs **1a**–**c**, **2a**–**c**, and **3a**–**c**.

CO-RMs **1a**–**c**, **2a**–**c**, and **3a**–**c** in Scheme 1 were obtained as solid crystals in a 40–85% yield range by treatment of intermediates P(1–3)a, P(1–3)b, and P(1–3)c with a slight excess (1.05 eq.) of dicobalt octacarbonyl (Co_2_(CO)_8_) in tetrahydrofuran (THF) as a solvent. In turn, the alkynyl precursors were synthesized by routine alkylation reactions of the appropriate and commercially available nucleophilic aniline, phenol, and thiol derivatives with propargyl bromide under basic reaction conditions (Scheme 1).

All final compounds were characterized by means of ^1^H-NMR, and their features were found to be in agreement with published data [29]. Elemental analyses account for >95% purity.

### 2.2. CO Release: A Computational Analysis

**Figure 3:**
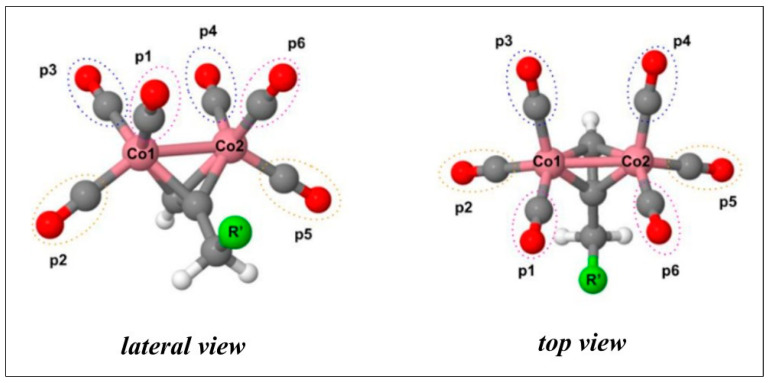
Lateral and top views of propargyl moiety. The aryl-X scaffold (R’) is represented by a green sphere. CO molecules, labeled as p1, p2, and p3, are bound to Co1, while p4, p5, and p6 coordinate the Co2 atom.

The geometries of CO-RMs **1a**–**c**, **2a**–**c**, and **3a**–**c** associated with their corresponding lowest energy levels were optimized at the B3LYP level of theory using the 6–31G* basis set for the cobalt atoms and cc-pVDZ for the remaining atoms (Figure S1). In agreement with previously reported data, the singlet state is the most stable electronic configuration [26]. All the derivatives present a RC_6_H_4_X—CH_2_ bond in a syn conformation, with the X atom located between the two p1 and p6 CO ligands (Figure 3). In addition, the geometrical parameters of the DCH moiety are not affected by the nature of the R substituent (Table S1).

The impact of X and R on backdonation was investigated by performing a perturbation theory energy analysis using the natural bond orbital (NBO) approach on the optimized DCH structures. This analysis allows for the estimation of the stabilization energy (E^ST^) associated with donor–acceptor interactions and, here, is specifically related to the interaction of the Co d-orbital electrons with π* and σ* orbitals of each CO molecule and Co-CO bonds, respectively. As reported in Table 1, the most relevant contribution to total E^ST^ is represented by E^ST^ (d → π* CO) associated with a π* backdonation effect. For each type of X derivative, an increase in the total E^ST^ was observed passing from –M (-NO_2_) to +M (-OCH_3_), indicating that R substituents endowed with +M features improve d–π* interactions and enhance the π* backdonation effect.

As shown in Table S2, the p2/p5 pair displays E^ST^ values spanning between 20.0 and 22.0 kcal mol^−1^, whereas for those of the other pairs lie within a range of 16.0–19.5 kcal mol^−1^. Such a trend agrees with the π* donation effect, which is higher for the former pair (i.e., shortest r_Co−CO_ bond) when compared to the others (Table S1).

Geometry optimizations for the LCo_2_(CO)_5_ structures revealed that the triplet spin state (^3^A) is more stable than the singlet (^1^A), at variance with the parent LCo_2_(CO)_6_ DCHs. Specifically, ^3^A was ~2–16 kcal mol^−1^ lower in free energy than ^1^A (Table S3), with the largest differences observed for p2 and p5. This trend reflects the known behavior of metal–CO complexes, where a reduction in the number of carbonyl ligands favors higher spin states [30].

Co_2_(CO)_5_ clusters have a specific geometry, which accounts for one CO bridging the Co atoms in a µ_2_ binding mode [31], whereas the remaining four CO ligands are disposed in a planar square-like coordination (Figures S2–S4). The µ_2_ binding mode was found for the ^3^A structures only, whereas the ^1^A geometries show all five COs in terminal binding mode, in agreement with Li et al. [32]. Interestingly, for **1b**[p1] and **1c**[p1], the N atom interacts with the antibonding orbitals of the closest CO ligand (E^ST^: 0.25 and 0.29 kcal mol^−1^, respectively), preventing the formation of µ_2_ coordination (Figure S2).

**Figure 4:**
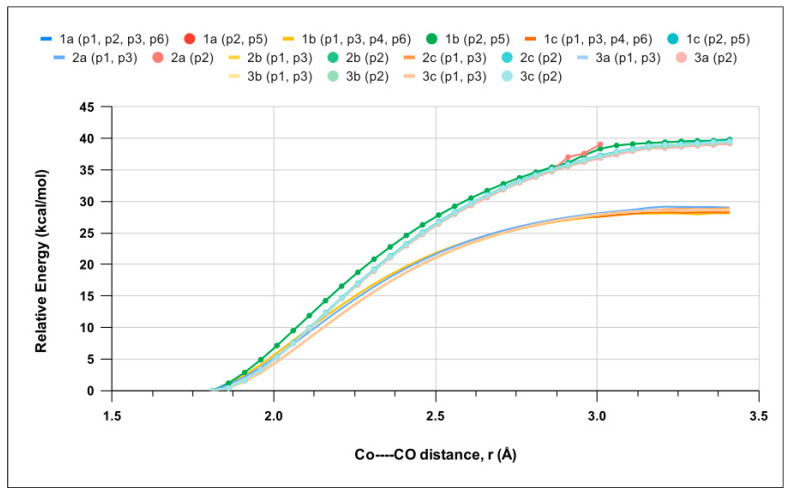
Electronic energy as a function Co–CO bond distance (r_Co−CO_) for the lowest-energy singlet among LCo_2_(CO)_6_ molecules **1a**, **1b**, **1c**, **2a**, **2b**, **2c**, **3a**, **3b**, and **3c** determined by relaxed scan calculations. Curves for p2 and p5 CO loss are highlighted by full circles. CO positions showing a behavior are grouped and presented in brackets.

Relaxed scan calculations were performed to investigate the kinetics of the first CO release by the singlet LCo_2_(CO)_6_ geometries and increases in the Co–CO bond distance (r_Co−CO_) for each CO position (p1-p6). Figure 4 shows that CO ligand release is barrierless, in analogy to CO-RM1 [25]. Therefore, the thermodynamics of the process is also expected to govern its kinetics (see the Discussion section). The potential energy curves (PEC) computed for the p1, p3, p4, and p6 positions are very similarand reach a plateau at ~28 kcal mol^−1^, whereas p2 and p5 peak at ~40 kcal mol^−1^. Such data clearly indicate that the release of CO is far easier at p1, p3, p4, and p6 and fit with the E^ST^ results (Table S2).

The above PECs were obtained by considering that CO release occurs on the same singlet surface. However, the most stable spin state of the LCo_2_(CO)_5_ products is a ^3^A triplet state, and a crossing between the singlet and triplet state might occur during the process. Therefore, we performed relaxed scan calculations along the triplet-state PEC for the **1b** complex, the results of which are shown in Figure S5. A crossing is observed between the two PECs for the p2 and p3 positions at a Co–CO distance of about 2.2 Å, with electronic energy estimated in the interval of 18–32 kcal mol^−1^ ca (Figure S5), the precise determination of which is demanding and beyond the scope of the present work.

Therefore, the thermodynamics of the CO release process was investigated by calculating the first Co–CO bond dissociation energy (FBDE) and the corresponding ΔG values considering the loss of CO molecules from each position starting from the lowest singlet LCo_2_(CO)_6_ and providing the lowest triplet LCo_2_(CO)_5_ states. Tables S4 and S5 report the most favorable FBDE and ΔG values for the considered set of compounds.

These data closely match (i.e., 25.0 ± 0.3 and 18.2 ± 0.4 kcal mol^−1^ for FBDE and ΔG, respectively), suggesting a comparable CO release capacity from the sampled compounds. As expected, due to the increase in entropy associated with dissociation processes, ΔG values are significantly lower than the FBDEs (Table S6). The FBDEs were also computed considering the singlet state of LCo_2_(CO)_5_ derivatives, obtaining more positive values, as reported in Table S7. The most favorable FBDE values are in the range of 28.0 ± 0.2 kcal mol^−1^, which is very close to the energy plateau obtained from scan calculations.

### 2.3. CO Release Assays

CO release was evaluated by means of the widely applied myoglobin (Mb) carbonylation assay, a spectrophotometric method that relies on Mb affinity for CO to afford the carbonylated myoglobin (Mb-CO) in a stoichiometric ratio [22,33].

Since an excess of acceptor (Mb) is known to favor CO release from CO-RMs, in our study, we first used a 1/1 CO-RM/Mb ratio to explore and compare the ligand-releasing properties under “unfavorable” conditions.

**Figure 5:**
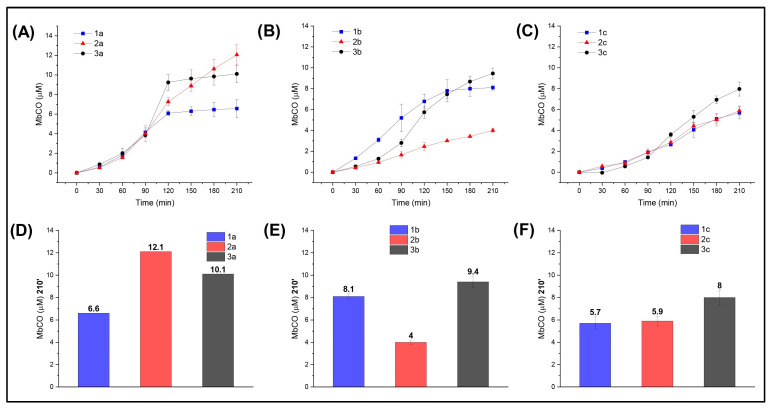
CO release profiles (such as Mb-CO formed over time) of compounds bearing –NO_2_ (**A**), –H (**B**), or –OCH_3_ (**C**) as substituents in the para (p) position of the aromatic ring; Mb-CO formed after 210′ incubation of myoglobin with –NO_2_ (**D**), –H (**E**), or –OCH_3_ (**F**) p-substituted compounds.

In line with the computational study, we investigated the compounds by comparing their CO release abilities depending on the R substituent and the heteroatom (X). For this purpose, the formed Mb-CO was determined at each time point up to 210 min after incubation of 20 μM deoxy-Mb with 20 μM CO-RM. Comparison of the CO-releasing profiles for the -NO_2_ (Figure 5A) and -OCH_3_ (Figure 5C) series up to 90 min showed that the nature of the heteroatom (X) has no influence in modulating the CO release rate and efficiency of the CO-RMs within each series, with CO release curves almost superimposable. However, after 90 min, S- and O-containing derivatives of the -NO_2_ series (compounds **2a** and **3a**) led to the formation of a higher concentrations of Mb-CO than the N analogue **1a**. In particular, compound **3a** provided rapid CO release up to 120′, reaching a plateau right after, whereas compound **2a** showed a slow but constant increase in CO release over time. This may be due to the higher propensity of S-containing compounds to promptly release CO when compared to its isosteric analogues, leading to the rapid formation of Mb-CO and an equilibrium being reached thereafter in an environment where little unbound Mb is available. As for the -OCH_3_ series, a slight impact of the X group was revealed only for S-containing derivative **3c** after 90′ of incubation.

The role played by the heteroatoms in modulating the CO release rate and efficiency was better revealed by the unsubstituted series (i.e., R = –H; Figure 5B), where the impact of the X group is not shaded by the R moiety. Specifically, X = N, S favor CO release, whereas X = O hampers the process.

In summary, the insertion of an electron-withdrawing R moiety seems to enhance CO release for S and O derivatives, but such an effect is not observed in the N-containing derivatives. The NO_2_-containing compounds are faster and more efficient releasers than the OCH_3_-containing compounds, regardless of the considered heteroatom (X). Overall, the electron-donating moiety -OCH_3_ strongly influences the CO release properties of the CO-RM in which it is inserted by turning it down and shadowing the contribution from X. The only exception is represented by O-containing derivative **2c**, where such an R group slightly improved CO release when compared to unsubstituted **2b**.

A common way to compare CO-RMs is by means of their T/X time, which is defined as the time (min) necessary for a CO-RM solution to produce a Mb-CO concentration equal to 1/6, 1/5, 1/4, 1/3, or 1/2 of its initial concentration. Figure S6 shows the T/X values calculated for the CO- RMs analyzed here. Overall, the fastest CO releasers are unsubstituted N-containing compound **1b** and the NO_2_-substituted compounds **3a**, **2a**, and **1a**.

**Figure 6:**
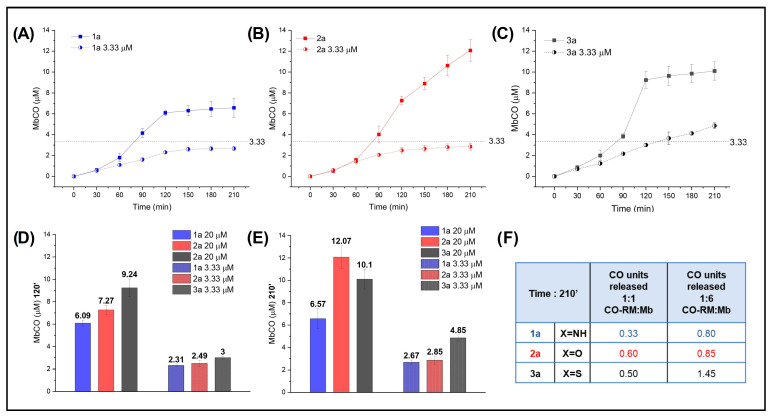
Mb-CO formed over time after the addition of 20 μM (1/1 CO-RM:Mb) (filled circles) and 3.33 μM (1/6 CO-RM:Mb) (half-filled circles) **1a** (**A**), **2a** (**B**), and **3a** (**C**) to 20 μM deoxymyoglobin in PBS; (**D**) Mb-CO formed after 120′ and (**E**) 210′ incubation of 20 μM deoxymyoglobin with 20 μM and 3.33 μM of **1a**, **2a**, and **3a**; (**F**) mole equivalent of CO released after 210′ incubation of 20 μM deoxymyoglobin with 20 μM and 3.33 μM of **1a**, **2a**, and **3a**.

With these data in hand, we analyzed NO_2_-substituted compounds **1a**, **2a**, and **3a** at different concentration ratios of acceptor (Mb) to determine any effect on CO release. The results are reported in Figure 6, where the mole equivalents of CO released by the CO-RMs at each concentration are also shown (Figure 6F).

An excess of acceptor (1/6 CO-RM:Mb, 3.33 μM) was confirmed to favor the release of CO from N- and O-containing derivatives, which were able to release almost 1 mole equivalent of CO when incubated with Mb in a 1/6 ratio. Interestingly, S-containing **3a** released 1.45 mole equivalent of CO, confirming that its intrinsic potential for CO release is higher than that of its isosteric analogues.

### 2.4. Determination of CO-RM Stability by Mass Spectrometry

The investigated CO-RM compounds were detected by MS either as protonated [M+H]^+^ or alkali metal [M+Na]^+^ and [M+K]^+^ adduct ions. The corresponding detected ions, *m*/*z* values, and CID fragments are summarized in Table S8. Unfortunately, no signals attributable to species **2b** and **2c** were recognized due to the lack of protonable sites.

Energy-resolved collision-induced dissociation (ER-CID) experiments were carried out on the bare cationized CO-RM analytes (**1b**, **1a**, and **1c**) for a comparative evaluation of the CO release ability in the absence of solvent, counterions, or matrix effects (e.g., sodium dithionite, DT). The CO loss profiles in the gas phase were obtained by increasing the collision energy values in the ion trap. Then, consecutive isolation and dissociation of the product ions allowed for determination of the removal of CO from the metal core. The recorded breakdown curves for [M+H]^+^ CO-RMs are reported in Figure S7A. The energy required to fragment 50% of the precursor ion (CID50%) decreased from 7.92 (**1b**) to 4.20 (**1c**), producing the main fragment at *m*/*z* 325, (C_9_H_3_O_6_Co_2_^+^), which then released carbon monoxide only at a relatively high collision energy as a secondary fragment (*m*/*z* 297). The presence of the –NO_2_ group (**1a**) favors CO release as the main process, even at minimal collision energy (CID50% = 3.79).

The measured breakdown curves for the [M-H^−^]^+^ ions, where only CO loss appears, confirm the role of the electron-withdrawing group in CID behavior. Figure S7B shows that CO loss occurs more promptly in **1c** (CID50% = 3.48) than in **1b** (CID50% = 10.23). For the latter, no fragmentation involving the organic portion was recorded. For the other CO-RMs listed in Table S8, it was not possible to achieve any appreciable quantitative curve due to the high lability of the complexes. Finally, the stability of N-containing derivatives (**1a**, **1b**, and **1c**) was tested in solution at 37 °C.

**Figure 7:**
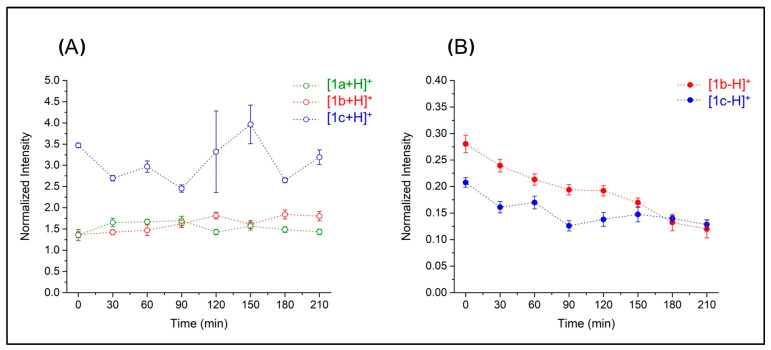
Values of the ion intensities of N-containing CO-RMs in a stability test normalized based on leucine–enkephalin ion intensity. Panel (**A**) reports intensities of protonated **1b**, **1a**, and **1c**, whereas panel (**B**) refers to the [**1b**-H^−^]^+^ and [**1c**-H^−^]^+^ species.

In all cases, the compounds are confirmed to be stable within 210 min (Figure 7A). Notably, the electron-donating group (–OCH_3_) in **1c** seems to positively influence the stability of the compound compared to unsubstituted derivative **1b** (Figure 7B). Partial initial reduction of both compound species was observed up to 30 min; then, **1c** became more stable than **1b** for the entire time frame of the experiment (210 min). A complete list of mass spectra depicting for the stability test conducted on **1c** is reported in Figure S8.

## 3. Discussion

The results of experimental and computational approaches converge, highlighting common aspects and providing complementary information on the CO release mechanism in CO-RM compounds. This is in relation to the effects of chemically significant structural variations in the drug sphere or in substituents. In our models, the introduction of R with ±M features appears to play the most significant role in the CO-release process. The stabilization energy (calculated using the NBO method), the free energy required for the first CO loss, the Mb assay, and the mass spectrometry stability studies on the N-containing derivatives all indicate that the +M effect hampers the release of CO. It is important to note that these results were obtained under very different conditions. The calculations were conducted with water as the solvent; the Mb assays were performed in a buffer solution containing sodium DT and Mb; and the mass spectrometry analysis was carried out on bare ions, which were transferred from acetonitrile solution to the gas phase via electrospray ionization. On one hand, this evidence confirms that the effect is dependent solely on the electronic structure of the investigated species. On the other hand, it demonstrates how the environment can modulate the resulting behavior. For instance, the theoretical study suggests that an increase in π* backdonation takes place when switching from –M to +M substituents. Such a finding is supported by the ER-CID experiments in the gas phases of protonated **1a**, **1b**, and **1c**. However, some deviations from this trend, mostly associated with longer release times, are observed in the Mb assays. Notably, the –NO_2_ DCH derivatives, which are associated with the strongest –M character, are generally the most efficient CO releasers.

Any modification of the X atom showed a relatively smaller impact on CO release. This effect is better captured computationally by the FBDE values, which align with the release trends observed in the Mb assay. The S derivatives release the most CO, except when R = –NO_2_. However, the computed FBDE values are very close in energy (25.0, 25.4, and 24.7 kcal mol^−1^ when X = O, NH, and S, respectively) and all within the accuracy range of the method. This is reflected in the experimental curves from the Mb assay, as the results for different molecules are almost superimposable or very close for the first 60–90 min. Environmental factors in the Mb assay can play additional roles with effects on the acquired results. Such an assay requires specific chemical conditions and reactants, such as sodium DT, to prevent Mb oxidation. Its decomposition in aqueous solution at pH ~7 takes place according to the following first-order reaction [34]:

2Na_2_S_2_O_4_ + H_2_O → 2NaHSO_3_ + Na_2_S_2_O_3_ (1)

The generation of the hydrogen sulfite ion under assay conditions contributes to the enhancement of CO release, as it is a binder of the CO-RM metal center itself [35]. The action of hydrogen sulfite or other nucleophilic sulfur species might explain the rapid rates of increase in CO release in the first 60–90 min, providing a rationale for the observed discrepancy between theoretical and experimental results of the Mb assay at the longest times. Early data from the Mb assay (i.e., those obtained up to 60 min when interference from side reactions is negligible) are highly valuable in estimating the energy of the first decarbonylation reaction (ΔE^exp^) directly from the experimental data by using the Arrhenius equation. The process is barrierless, and the energy barriers obtained in such a way provide a good estimation of Co–CO binding energy, which is useful for comparison with data obtained by relaxed scan calculations relative to the first release event [25]. By doing so, we assume that (i) all released CO interacts with Mb under the experimental conditions and (ii) CO release occurs by means of a first-order reaction. The corresponding values are reported in Table S9. The average ΔE^exp^, considering all sets of molecules, is 24.9 ± 0.3 kcal mol^−1^, a value in excellent agreement with the average FBDE of 25.0 ± 0.3 kcal mol^−1^ computed considering the product LCo_2_(CO)_5_ in its triplet state, confirming the spin transition from singlet to triplet of the dicobalt center upon loss of the first CO molecule. A previous theoretical study completely based on the singlet state for both H_2_C_2_Co_2_(CO)_6_ and H_2_C_2_Co_2_(CO)_5_ did, indeed, predict an FBDE value of 30.1 kcal mol^−1^ at the B3LYP/DZP level of theory [32], which is very similar to the FBDE obtained here when considering the singlet state for LCo_2_(CO)_5_. Although we provided a very rough estimate, the crossing between the singlet and triplet PECs was shown to occur at electronic energies below 30 kcal mol^−1^ (the corresponding enthalpy is estimated to be lower), making the spin-forbidden transition the energetically favored path, as confirmed by the ΔE^exp^ data from the Mb assay. The accurate computational determination of the crossing points and of the transition energies requires more raffinate computational techniques [36,37], like CASSCF and TD-DFT, and is currently in progress.

Mb assay results at longer release times are affected by environmental effects and can, therefore, help to shed some light on CO release mechanism under biological conditions—a much-debated issue. Several authors have reported that some CO-RMs, such as those belonging to the DCH family, release CO only in the presence of an acceptor such as Mb [38,39]. To verify this, the results of the MS experiments shown in Figure 7 can be useful; the stability of protonated derivatives of aryl-X-propargyl DCH ligands **1a**, **1b**, and **1c** was probed in acetonitrile solution in the absence of Mb at 37 °C. The figure shows that the NH derivatives are very stable, and no significant CO release could be recorded throughout the whole 210 min observation time (the same used for the Mb assay). This outcome agrees with the computed ΔG values for the first decarbonylation reaction, indicating that this is a largely endergonic process and suggesting that, in solution, spontaneous CO release from DCH molecules is not likely to happen. Therefore, an efficient CO acceptor can play a relevant role in CO release. Gill and co-workers investigated the thermochemistry of the interaction between CO and several Mb isoforms by performing calorimetric experiments [40]. They reported that the reaction between horse Mb and CO is widely exothermic, with the entropy term being unfavorable, so that the Mb–CO association (Equation (2)) is exergonic overall, with ΔG° = −9.5 kcal mol^−1^.

MbFe^II^ + CO → MbFe^II^CO                ΔG° = −9.5 kcal mol^−1^ (2)

LCo_2_(CO)_6_ → LCo_2_(CO)_5_ + CO        ΔG° = +18.2 kcal mol^−1^ (3)

LCo_2_(CO)_6_ + MbFe^II^ → LCo_2_(CO)^5^ + MbFe^II^CO        ΔG° = +8.7 kcal mol^−1^ (4)

Starting from this value, we can evaluate the effect of the Mb acceptor on the thermodynamics of CO release for the investigated cobalt-based CO-RMs (Equation (4)) by coupling the breaking of the Co–CO bond in our dicobalt CO-RMs (Equation (3)), with an average ΔG° = +18.2 kcal mol^−1^ and carbonylation of Mb (Equation (2)).

The resulting ΔG° = +8.7 kcal mol^−1^ indicates that the presence of Mb does increase the amount of released CO. However, the total reaction remains endergonic under standard conditions, suggesting that DCH derivatives cannot release a significant amount of the first CO molecule, in agreement with the Mb assay results obtained with a 1/1 CO-RM/Mb ratio (Figure 5). However, a large excess of Mb, achieved by the experimental 1/6 CO-RM/Mb ratio conditions shown in Figure 6, can dramatically shift the equilibrium in Equation (4) towards Mb-CO formation, leading to a quantitative CO release. Such a situation may occur in specific biological environments where many chemical species can accept/react with CO, promoting a significant CO release. The target selectivity of cobalt-based CO-RMs can, thus, be improved by combining this feature with a specific drug delivery system [41].

## 4. Materials and Methods

### 4.1. Chemistry

Anhydrous solvents and all reagents were purchased from Sigma-Aldrich (Milan, Italy), Alfa Aesar (Milan, Italy), and TCI (Milan, Italy). All reactions involving air- or moisture-sensitive compounds were performed under a nitrogen atmosphere using dried glassware and syringe techniques to transfer solutions. Nuclear magnetic resonance spectra (^1^H-NMR: 400 MHz; ^13^C-NMR: 100 MHz) were recorded in DMSO-d6 or CDCl_3_ using an Avance III 400 MHz spectrometer (Bruker, Milan, Italy), as reported in Figures S9–S14. Chemical shifts are reported in parts per million (ppm), and the coupling constants (J) are expressed in Hertz (Hz). Splitting patterns are designated as follows: s, singlet; d, doublet; t, triplet; m, multiplet; brs, broad singlet. Analytical thin-layer chromatography (TLC) was carried out on F-254 silica gel plates (Merck, Milan, Italy). The melting points of solid compounds were measured on a MEL-TEMP capillary melting point apparatus from ambient temperature up to 300 °C.

#### 4.1.1. General Procedure for the Synthesis of Derivatives P(1–3)a, P(1–3)b, and P(1–3)c 

As summarized in Scheme 1, to a solution of starting material (0.5 g, 1.0 eq.) in dry DMF (4 mL), K_2_CO_3_ (1.5 eq.) was added, and the mixture was stirred for 15 min. Then, propargyl bromide 80% in toluene (1.5 eq.) was added dropwise. The mixture was stirred at the appropriate temperature, depending on the substrate used, until consumption of the starting material (TLC monitoring). The reaction mixture was quenched with H_2_O or crushed ice (20 mL) and extracted with EtOAc (3 × 25 mL). The organic layer was washed with brine (4 × 15 mL), dried over Na_2_SO_4_, filtered off, and concentrated under vacuum to yield a residue that was purified by silica gel column chromatography (EtOAc/n-Hexane).

#### 4.1.2. General Procedure for the Synthesis of CO-RM Derivatives 

As summarized in Scheme 1, the appropriate alkynyl derivative (0.1 g, 1.0 eq.) was dissolved in THF (5 mL)m and dicobalt octacarbonyl (1.05 eq.) was added. The black mixture was stirred at r.t. for 20–40 min (TLC monitoring). Then, SiO_2_ was added, and the solvent was removed under vacuum to yield a black solid residue, which was purified by silica gel column chromatography eluting with the appropriate mixture of EtOAc in n-hexane (*v*/*v* in %) to afford the desired compounds as red/black solids [23].

4-Nitro-N-(prop-2-yn-1-yl)aniline hexacarbonyldicobalt (**1a**) was synthesized according to the general procedure B using P1a as a substrate as follows: purified eluting with EtOAc/n-Hex 20% *v*/*v*; 56% yield; silica gel TLC Rf = 0.43 (EtOAc/n-Hex 20% *v*/*v*); δH (400 MHz, DMSO-d6): 4.66 (2H, d, J = 5.4), 6.78–6.67 (3H, m), 7.94 (1H, t, J = 5.9), 8.02 (2H, t, J = 8.8). Elemental analysis: calculated (%) C, 38.99; H, 1.75; N, 6.06; found C, 39.02; H, 1.78; N, 6.01. Experimental values are in agreement with reported data [29].

1-Nitro-4-(prop-2-yn-1-yloxy)benzene hexacarbonyldicobalt (**2a**) was synthesized according to general procedure B using P2a as a substrate. Purified eluting was performed with EtOAc/n-Hex 7% *v*/*v* as follows: 86% yield; silica gel TLC Rf = 0.55 (EtOAc/n-Hex 7% *v*/*v*); m.p. 108–109 °C; δH (400 MHz, DMSO-d6): 5.51 (2H, s), 6.85 (1H, s), 7.20–7.22 (2H, d, J = 8.8), and 8.25–8.27 (2H, d, J = 8.8). Elemental analysis: calculated (%) C, 38.91; H, 1.52; N, 3.02; found C, 38.96; H, 1.58; N, 2.99.

4-Nitrophenyl(prop-2-yn-1-yl)sulfane hexacarbonyldicobalt (**3a**) was synthesized according to general procedure B using P3a as a substrate. Purified eluting was performed with EtOAc/n-Hex 7% *v*/*v* as follows: 86% yield; silica gel TLC Rf = 0.75 (EtOAc/n-Hex 10% *v*/*v*); δH (400 MHz, DMSO-d6): 4.78 (2H, s), 6.64 (1H, s), 6.61–6.63 (2H, d, J = 8.4), and 8.16–8.18 (2H, d, J = 8.4). Elemental analysis: calculated (%) C, 37.60; H, 1.47; N, 2.92; found C, 37.63; H, 1.49; N, 2.94.

N-(Prop-2-yn-1-yl)aniline hexacarbonyldicobalt (**1b**) was synthesized according to general procedure B using P1b as a substrate. Purified eluting was performed with EtOAc/n-Hex 5% *v*/*v* as follows: 56% yield; silica gel TLC Rf = 0.43 (EtOAc/n-Hex 20% *v*/*v*); δH (400 MHz, DMSO-d6): 4.49 (2H, d, J = 6.6), 6.32 (1H, t, J = 6.5), 6.63–6.51 (4H, m), 7.08 (2H, t, J = 7.7); δC (100 MHz, DMSO-d6): 44.9, 74.1, 95.2, 112.32, 116.1, 129.8, 147.3, and 199.9. Elemental analysis: calculated (%) C, 43.19; H, 2.17; N, 3.36; found C, 43.22; H, 2.15; N, 3.31.

Prop-2-yn-1-yloxybenzene hexacarbonyldicobalt (**2b**) was synthesized according to general procedure B using P2b as substrate with an 81% yield. Purified eluting was performed with EtOAc/n-Hex 3% *v*/*v* as follows: 56% yield; silica gel TLC Rf = 0.43 (EtOAc/n-Hex 3% *v*/*v*); δH (400 MHz, DMSO-d6): 5.33 (2H, s), 6.84 (1H, s), 7.01 (3H, m), and 7.35 (2H, t, J = 8.0). Elemental analysis: calculated (%) C, 43.09; H, 1.93; found C, 43.04; H, 1.95. Experimental values are in agreement with reported data [29].

Phenyl(prop-2-yn-1-yl)sulfane hexacarbonyldicobalt (**3b**) was synthesized according to general procedure B using P3b as a substrate. Purified eluting was performed with EtOAc/n-Hex 7% *v*/*v* as follows: 55% yield; silica gel TLC Rf = 0.95 (EtOAc/n-Hex 10% *v*/*v*); δH (400 MHz, DMSO-d6): 4.60 (2H, brs), 6.52 (1H, brs), and 6.40 (5H, brs). Elemental analysis: calculated (%) C, 41.50; H, 1.86; S, 7.38; found C, 41.53; H, 1.82; S, 7.40.

4-Methoxy-N-(prop-2-yn-1-yl)aniline hexacarbonyldicobalt (**1c**) was synthesized according to general procedure B using P1c as a substrate. Purified eluting was performed with EtOAc/n-Hex 5% *v*/*v* as follows: 40% yield; silica gel TLC Rf = 0.37 (EtOAc/n-Hex 5% *v*/*v*); δH (400 MHz, DMSO-d6): 3.63 (3H, s), 4.46 (2H, d, J = 6.6), 5.92 (1H, t, J = 6.3), 6.58 (3H, m), and 6.74 (2H, d, J = 8.7). Elemental analysis: calculated (%) C, 42.98; H, 2.48; N, 3.13; found C, 43.01; H, 2.49; N, 3.17.

1-Methoxy-4-(prop-2-yn-1-yloxy)benzene hexacarbonyldicobalt (**2c**) was synthesized according to general procedure B using P2c as a substrate. Purified eluting was performed with EtOAc/n-Hex 7% *v*/*v* as follows: 73% yield; silica gel TLC Rf = 0.85 (EtOAc/n-Hex 10% *v*/*v*); δH (400 MHz, DMSO-d6): 3.71 (3H, s), 5.23 (2H, s), 6.78 (1H, s), and 6.91 (4H, brs). Elemental analysis: calculated (%) C, 42.89; H, 2.25; found C, 42.91; H, 2.23.

4-Methoxyphenyl(prop-2-yn-1-yl)sulfane hexacarbonyldicobalt (**3c**) was synthesized according to general procedure B using P3c as a substrate. Purified eluting was performed with EtOAc/n-Hex 8% *v*/*v* as follows: 56% yield; silica gel TLC Rf = 0.90 (EtOAc/n-Hex 10% *v*/*v*); δH (300 MHz, DMSO-d6): 3.73 (3H, bs), 4.41 (2H, bs), 6.38 (1H, s), 6.92 (2H, bs), 7.37 (2H, bs). Elemental analysis: calculated (%) C, 41.40; H, 2.17; S, 6.91; found C, 41.42; H, 2.15; S, 6.94.

### 4.2. CO Release Assay

All reagents were of analytical grade and purchased from Merck (Milan, Italy). Gaseous CO was obtained from Rivoira (Milan, Italy). A Shimadzu UV1900 UV-Vis Spectrophotometer operated in the range of 275 to 700 nm at a scanning rate of 200 nm/min was used to record UV-Vis absorption spectra in a disposable plastic cuvette (path length, 0.44 cm). The second-derivative spectra were generated by using Origin Lab software (https://www.originlab.com/, accessed on 27 October 2024), and the Savitzky–Golay method was applied using 25 data points for the differentiation process. Neither an increase nor a decrease in the number of points caused changes in the wavelength or in the bandwidth. Lyophilized horse heart Mb was dissolved in phosphate-buffered saline flushed with N_2_ (PBS, 0.01 M and pH 7.4 to a 20–22 μM final concentration). Then, 2 mL of this freshly prepared stock solution was placed in a cuvette to record the UV-Vis absorption spectrum of met-Mb. Next, the solutions were divided into two halves; 10 μL of sodium dithionite (30 mg/mL) was added to the first half (reference), and the UV-Vis spectrum of deoxy-Mb was registered. After that, the solution was flushed with CO gas, and the Mb-CO spectrum was acquired. Sodium dithionite was added to the second half (sample), and a spectrum was recorded. Afterwards, a CO-RM DMSO solution was added to a final CO-RM concentration of 20 μM and gently mixed. The solution was covered with 300 μL of light mineral oil to avoid the escape of CO and oxygenation of Mb, and the absorption spectrum was recorded at t = 0. Spectra were acquired every 30 min for 210 min, keeping the sample at 37 °C. When necessary, a freshly prepared sodium dithionite solution was added. After 210 min, the total Mb concentration at the end of the assay was determined by flushing the sample with CO gas. Each experiment was replicated three times, and the mean of the three results was calculated for each time point. Therefore, data were expressed as mean ± SEM. The Mb-CO concentration at each time point was determined as previously reported [22].

### 4.3. Mass Spectrometry CO-RM Stability Study

All solvents were analytical-grade, commercial products (Merck, Milan, Italy) and used as received. Stock solutions of analytes were prepared in acetonitrile at 1.0 mg mL^−1^ and diluted in the same solvent to a final concentration of 50 µM with 0.1% formic acid added as an ionization dopant. The mass spectra were obtained by using two different instruments. A BioApex Fourier transform ion cyclotron resonance (FT-ICR) [42] mass spectrometer (Bruker Daltonics GmbH, Bremen, Germany) equipped with an electrospray source (ESI) was operated for accurate mass determinations. The FT-ICR mass spectra were internally frequency-to-*m*/*z*-calibrated compared to ions of known elemental composition. All mass spectra were recorded in positive polarity mode (ESI+) by continuously infusing the analyte solutions at a rate of 140 μL h^−1^. In the second setup, energy-resolved collision-induced dissociation (ER-CID) experiments were carried out on mass-selected ions with He as a collision gas using a linear ion trap mass spectrometer (LTQ XL, Thermo Scientific, Waltham, MA, USA). The survival yield curves were generated by plotting the survival yield obtained according to Equation (5) as a function of the applied collision energy (CE) as follows [43]:

Survival Yield = I_p_/(I_p_ + ΣI_f_) (5)

In Equation (5), I_p_ and I_f_ are the intensity of the precursor and product ions, respectively. The CE values necessary to produce 50% precursor ion dissociation (CID50%) were extrapolated from the fit and employed to evaluate the relative stabilities of CO-RM ions. CO-RM stabilities were also assessed in acetonitrile solution. To mimic the physiological conditions, the sampled CO-RM solutions were stored at 37 °C, then assayed by mass spectrometry at regular time intervals, i.e., every 30 min up to 210 min. To assess the change in ion abundances over time, a calibrated solution of leucine–enkephalin 1.5 × 10^−5^ M was added as a stable internal standard, and its protonated ion (*m*/*z* 556.3) was used as a reference peak for quantitative comparison. To evaluate the changes in CO-RM abundances over time, raw data were normalized by sum; then, the signal/standard intensity ratio was obtained. All experiments were performed in triplicate.

### 4.4. Theoretical Calculations

The molecular geometries were built starting from the dicobalt(0) μ-C_2_H_2_(CO)_6_-optimized structure published by Platts et al. [44]. Several conformers were evaluated by systematic rotation of the main rotatable torsional angles. Each derivative can release up to six CO molecules, whose positions are here referred to as p1 to p6, as shown in Figure 3. CO molecules in p1, p2, and p3 are bound to Co1, while p4, p5, and p6 coordinate Co2.

The release of the first CO molecule is described by the following reaction:

LCo_2_(CO)_6_ → LCo_2_(CO)_5_ + CO (6)

where L represents the aryl propargyl scaffold. For each CO-RM reported in Figure 2, we separately considered the loss of each of the six CO molecules (p1–p6), and the resulting dicobalt(0)pentacarbonyl derivative, (LCo_2_(CO)_5_) is referred to by adding the position of the lost CO to the name of parent molecule, e.g., **a1**[p1]. The geometries of aryl-X-propargyl and the corresponding pentacarbonyl derivatives were minimized in the gas phase by adopting the same level of theory as that used by Platts et al. [44], consisting of the unrestricted density functional theory (UDFT) with a 6–31G* basis set [45] for the cobalt atoms and cc-pVDZ [46] for the remaining atoms, with the B3LYP hybrid functional [47]. Several spin states were assessed for both systems.

IR frequency calculations were carried out on the optimized geometries at the same level of theory to compute thermochemical parameters (zero-point energy (ZPE) and thermal corrections, entropies, and free energies at 298.15 K). Enthalpy values in water were obtained by performing single-point calculations with the conductor-like polarizable continuum model (cpcm) [48,49], then applying enthalpy thermal corrections computed in the gas phase.

The first Co–CO bond dissociation energy (FBDE), i.e., the enthalpy variation associated with the release of the first CO molecule (Equation (7)) [50], was computed for each species at each position (p1–p6) as follows:

FBDE = ∆H = {H[CO] + H[LCo_2_(CO)_5_]} − H[LCo_2_(CO)_6_] (7)

Gibbs free energy values were computed in water by including the solvation entropy, as determined by adopting the Werz approach [51,52], for which a solute dissolved in water loses a constant fraction of its entropy in the gas phase according to the following equation:

ΔS_solvation_ = −0.46(S_gas_ − 14.3 cal mol^−1^ K^−1^) − (6.32 cal mol^−1^ K^−1^) (8)

∆G values for CO release process were computed as follows:

∆G = {G[CO] + G[LCo_2_(CO)_5_]} − G[LCo_2_(CO)_6_] (9)

For each minimized DCH structure, a natural bond orbital (NBO) analysis was performed to estimate the impact of the backdonation effects on the Co–CO bond strength by computing the stabilization energy (E^ST^) associated with the delocalization of the d-orbital lone pair of Co (donor NBO) and the antibonding molecular orbitals (π* and σ*of CO and Co–CO (acceptor NBOs), respectively. 

To investigate the kinetic mechanism of CO release, relaxed scan calculations were performed along the bond-breaking reaction coordinate (r_Co−CO_), as proposed by Cavalli and co-workers [25], using a step of 0.05 Å for a total of 45 steps. NBO analysis and relaxed scan calculations were performed in water at the same level of theory reported above. All calculations were performed using Gaussian 09 software [53].

## 5. Conclusions

In this joint experimental and computational work, we designed a small, homogeneous series of DCH CO-RM derivatives differing by either R substituents or by the atom connecting the CO-RM moiety to the drug sphere, with the aim of analyzing the CO release mechanism and the electronic effect modulating it.

CO release experiments based on Mb-CO formation and the computed bond dissociation energy for the loss of the first CO molecule indicate that such a process is an endothermic process overall. This result agrees remarkably well with an energy value of ~25 kcal mol^−1^ when the spin transition from the reactant singlet state to the more stable product triplet state is considered. This value makes a spontaneous CO release process unlikely. On the other hand, the experimental data, together with theoretical indications, suggest that the presence of a specific acceptor (as Mb) or reactant, as might easily occur in biological media, can strongly enhance CO release.

From the structural standpoint, it was found that the impact of R substituents is more relevant than that of X-connecting atoms. Such an effect was detected under all experimental and computational approaches considered herein. An R moiety endowed with a mesomeric electron-withdrawing effect (−M), such as -NO_2_, facilitated CO release by reducing the interaction between d electrons of Co and the non-bonded π* orbital of CO groups (backdonation effect). Therefore, chemical modification of the drug sphere by including one or more functional groups with ±M effect can help modulate CO release. The nature of the atom connecting the propargyl tail and the aromatic ring can affect, although in a less predictable fashion, the first Co–CO bond dissociation energy following the order of O > NH > S, with more favorable values obtained when an S atom bridges the aryl and propargyl groups. The elucidation of the multiple issues involved in CO release from Co-based CO-RMs can help to direct their design to enhance their specific properties.

## Figures and Tables

**Table 1:** Stabilization energy values in kcal mol^−1^ and E^ST^ (d → π* CO) and E^ST^ (d → σ* Co-CO) of donor–acceptor interactions corresponding to the delocalization of the Co d orbital lone pair on the π* and σ* empty orbitals of CO moieties and on the Co-CO bonds, respectively.

Molecule	E^ST^ (d → π* CO)	E^ST^ (d → σ* Co-CO)	Total E^ST^
**1a**	106.0	4.3	110.3
**1b**	106.5	4.3	110.9
**1c**	108.8	4.8	113.6
**2a**	109.8	4.3	114.2
**2b**	110.6	4.7	115.3
**2c**	110.7	5.1	115.8
**3a**	107.4	4.7	112.1
**3b**	108.1	4.8	112.9
**3c**	108.4	4.8	113.2

## Data Availability

The optimized geometries of the CO-RMs investigated in this work are available upon request.

## Supplementary Materials

The following supporting information can be downloaded at: https://www.mdpi.com/article/10.3390/ijms252111644/s1.
