# Supplementary material for: Carbon Monoxide Release from Aryl-Propargyl Dicobalt(0)Hexacarbonyl Derivatives: A Computational and Experimental Study
Source: Int J Mol Sci. 2024 Oct 30;25(21):11644. doi: 10.3390/ijms252111644 (PMC11546923; doi:10.3390/ijms252111644)
Supplement: Supplementary file 1 [file ijms-25-11644-s001.zip › ijms-3287206-supplementary.pdf]

Supporting Information

Carbon monoxide release from the arylpropargyl dicobalt(0)hexacarbonyl derivatives: a computational and experimental study

Roberto Paciotti [1,*], Cecilia Coletti [1,*], Emanuela Berrino [2], Francesca Arrighi [2], Alessandro Maccelli [3], Alba Lasalvia [2], Maria Elisa Crestoni [2], Daniela Secci [2], Simone Carradori [1], Claudiu T. Supuran [4] and Fabrizio Carta [4]

1 Department of Pharmacy, "G. d'Annunzio" University of Chieti-Pescara, Via dei Vestini 31, 66100 Chieti, Italy; simone.carradori@unich.it
2 Department of Drug Chemistry and Technologies, Sapienza University of Rome, P.le A. Moro 5, 00185 Rome, Italy; emanuela.berrino@gmail.com (E.B.); francesca.arrighi@uniroma1.it (F.A.); alba.lasalvia@uniroma1.it (A.L.); mariaelisa.crestoni@uniroma1.it (M.E.C.); daniela.secci@uniroma1.it (D.S.)
3 National Centre for the Control and Evaluation of Medicines, Chemical Medicines Unit, Istituto Superiore di Sanità, Viale Regina Elena 299, 00161 Rome, Italy; a.maccelli@gmail.com
4 NEUROFARBA Department, Sezione di Scienze Farmaceutiche e Nutraceutiche, University of Florence, Sesto Fiorentino, 50019 Florence, Italy; claudiu.supuran@unifi.it (C.T.S.); fabrizio.carta@unifi.it (F.C.)
* Correspondence: r.paciotti@unich.it (R.P.); cecilia.coletti@unich.it (C.C.)

Table of contents

DFT calculations (Figures S1-S5) pages S2 - S6

CO-release studies (Figure S6) page S7

Mass Spectrometry (Figures S7, S8) pages S8, S9

NMR spectra of sampled compounds (Figures S9-S14) pages S10 - S14

Tables S1 – S9 pages S14 - S20

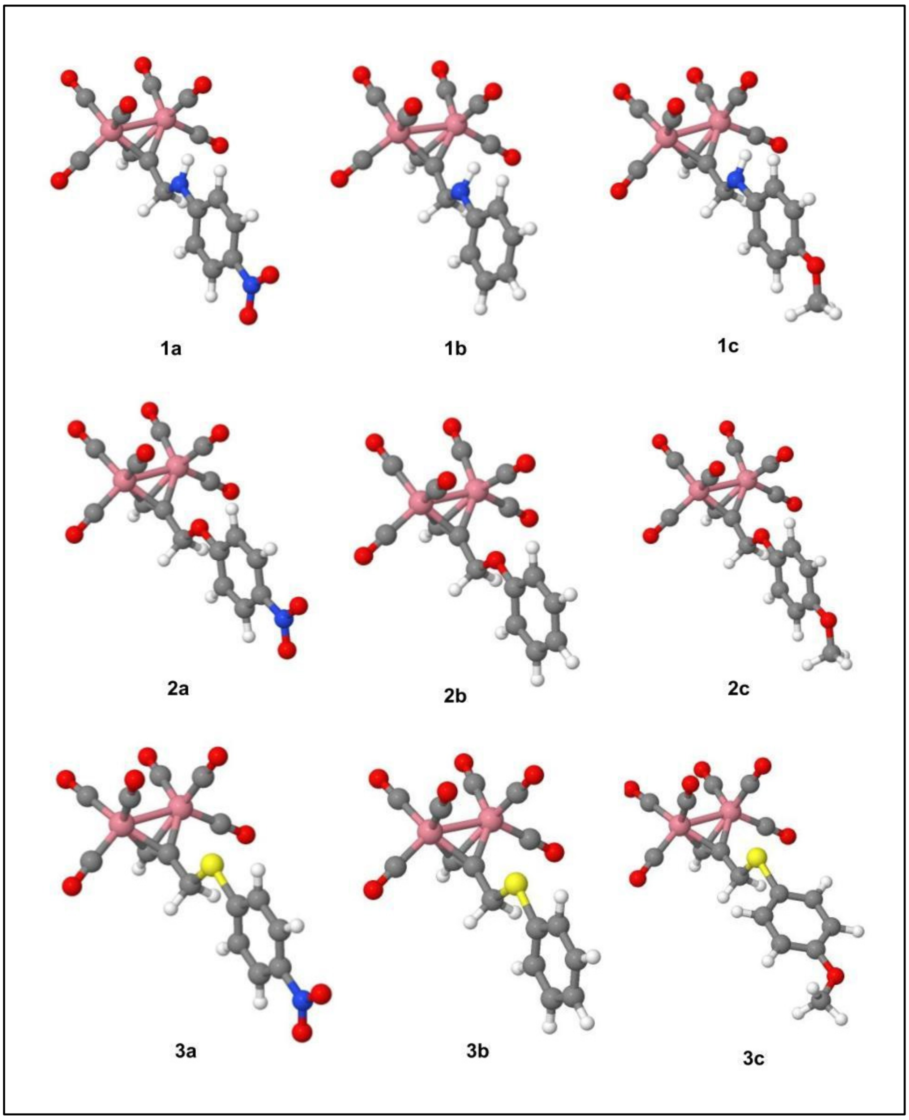

Figure S1. Geometries of **1a**, **1b**, **1c**, **2a**, **2b**, **2c**, **3a**, **3b** and **3c** optimized at B3LYP level of theory with 6-31G*/cc-pVDZ basis set.

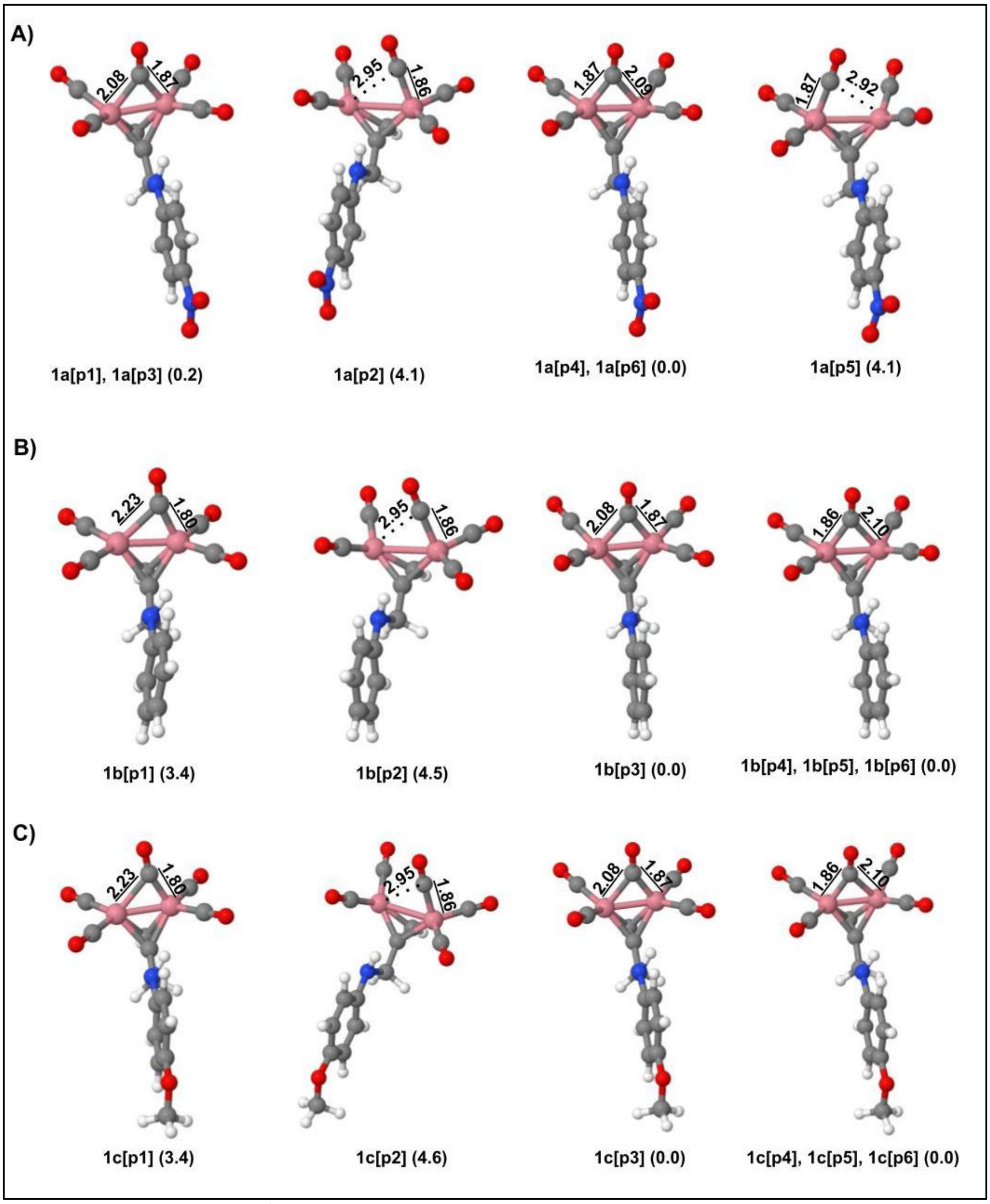

Figure S2. Geometries of ^{3}A $LCo_2(CO)_5$ derivatives for A) **1a**, B) **1b** and C) **1c** optimized at the B3LYP level of theory with 6-31G*/cc-pVDZ basis set. The relative free energy values with respect to the most stable isomer are reported in parenthesis in kcal mol^{-1}. The leaving CO group position is reported in square brackets.

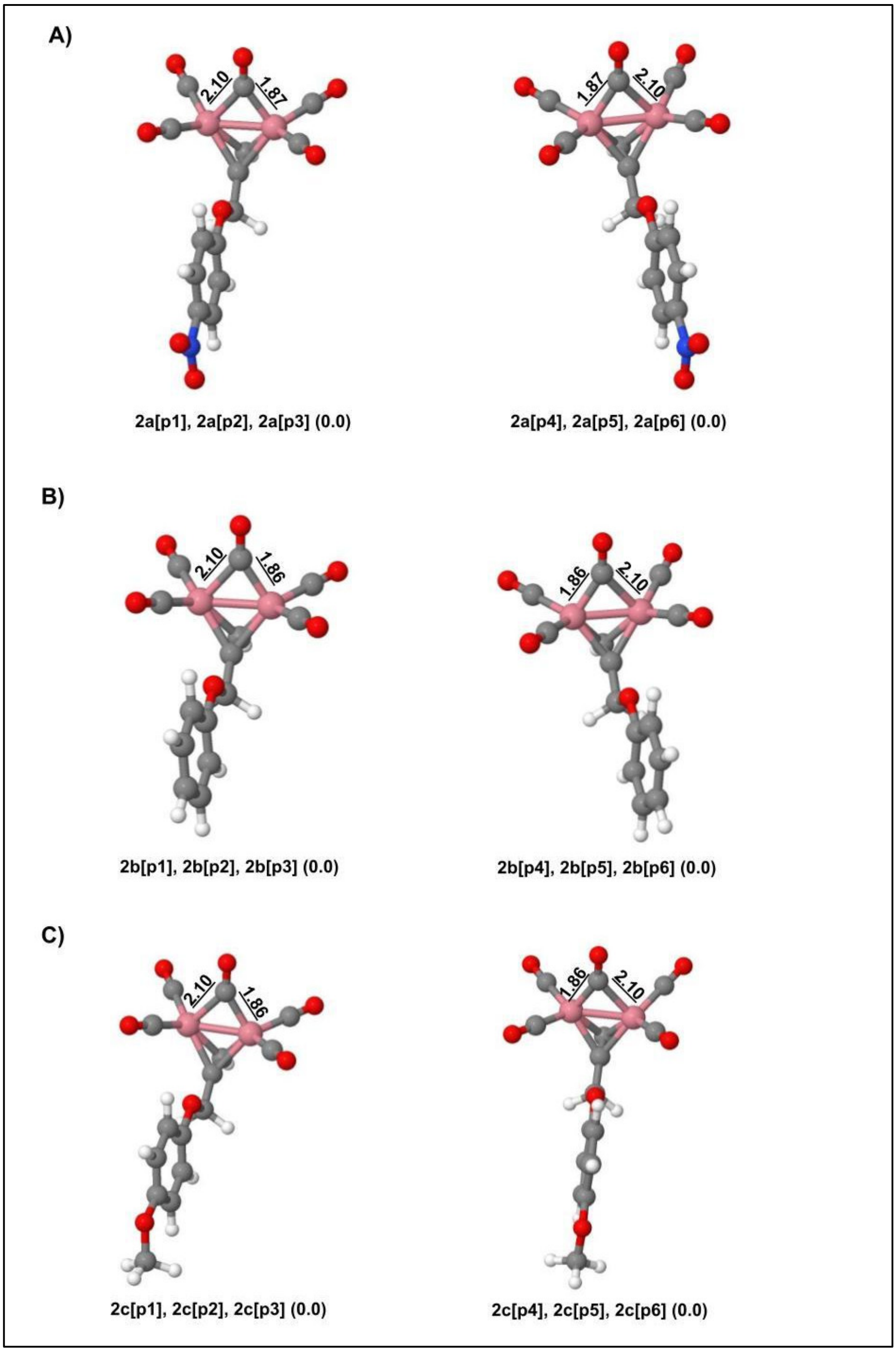

Figure S3. Geometries of ^{3}A $LCo_2(CO)_5$ derivatives for A) **2a**, B) **2b** and C) **2c** optimized at B3LYP level of theory with 6-31G*/cc-pVDZ basis set. The relative free energy values with respect to the most stable isomer are reported in parenthesis in kcal mol^{-1}. The leaving CO group is reported in square brackets.

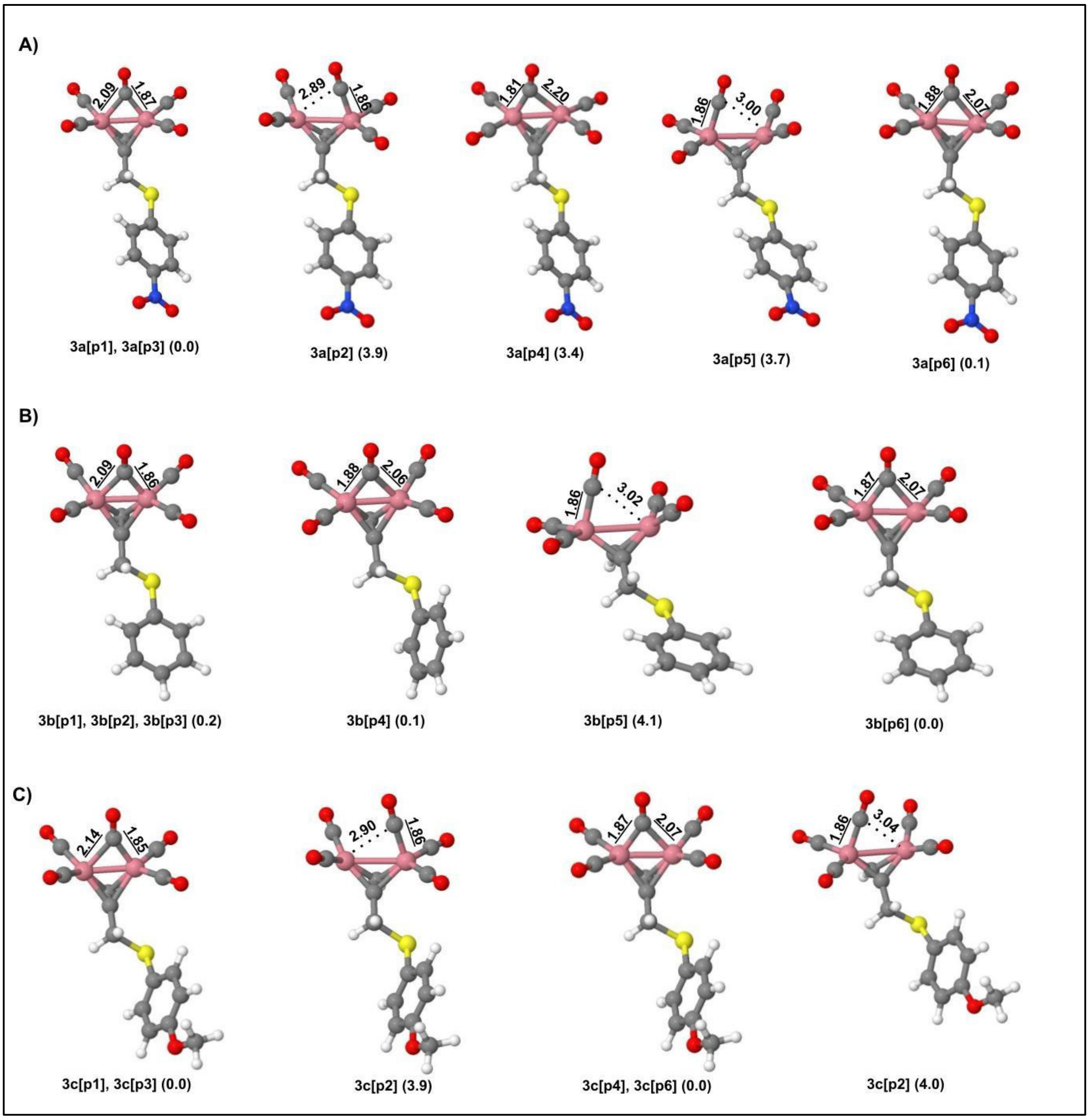

Figure S4. Geometries of ^{3}A $LCo_2(CO)_5$ derivatives for A) **3a**, B) **3b** and C) **3c** optimized at the B3LYP level of theory with 6-31G*/cc-pVDZ basis set. The relative free energy values with respect to the most stable isomer are reported in parenthesis in kcal mol^{-1}. The leaving CO group is reported in square brackets.

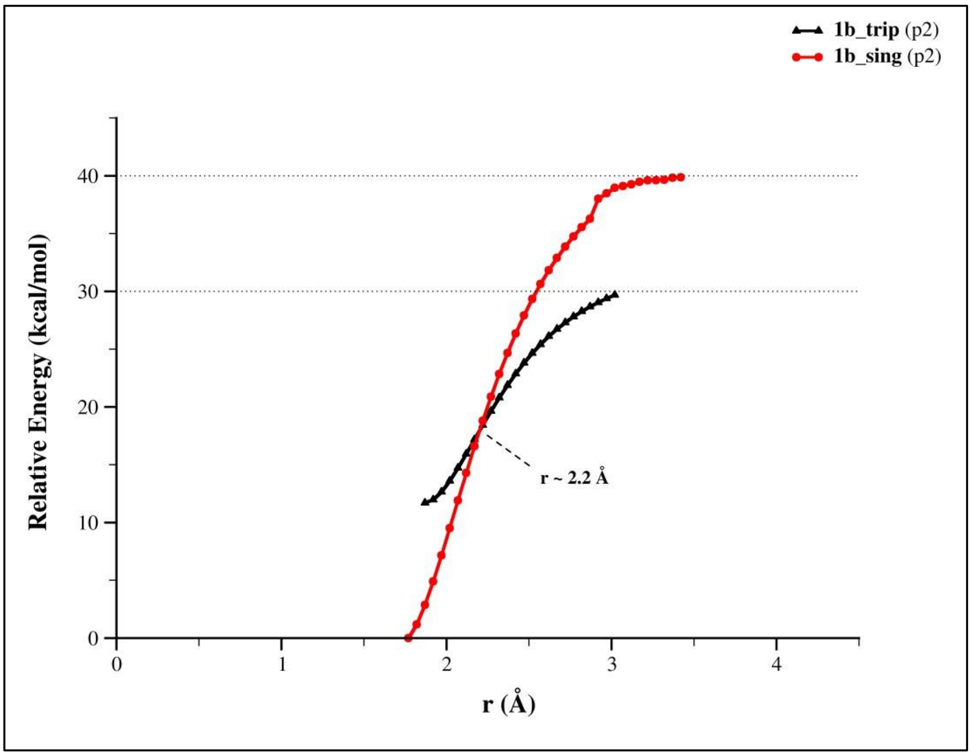

Figure S5. Relative electronic energies of N-aryl-propargyl derivative, **1b**, on the ^{1}A (red) and ^{3}A (black) spin states as a function of the Co-CO[p2] distance. The scan step of $r_{Co\text{-}CO}$ coordinate is 0.05 Å for a total of 45 steps. Relaxed scan calculations were performed at B3LYP level of theory with 6-31G*/cc-pVDZ basis set in methanol (CPCM). The two curves cross at about ~2.2 Å and at an energy of 18 kcal mol^{-1}. The crossing between singlet and triplet states occurring during the CO release process were evaluated at the relaxed geometry of each of two states. For sake of clarity, it is necessary to state that the obtained values are better considered as descriptors of the lowest limit for the effective crossing event. The upper limit can be obtained by adding the conformational penalty needed for the transition from singlet to the triplet relaxed geometry, which we calculated as ~14 kcal mol^{-1}. The crossing associated electronic energy is therefore allocated in the interval 18-32 kcal mol^{-1}, thus far more favorable than the energy required to break the Co-CO bond remaining on the singlet potential energy curve. A more accurate determination of such a value is beyond the scope of the present investigation.

A)

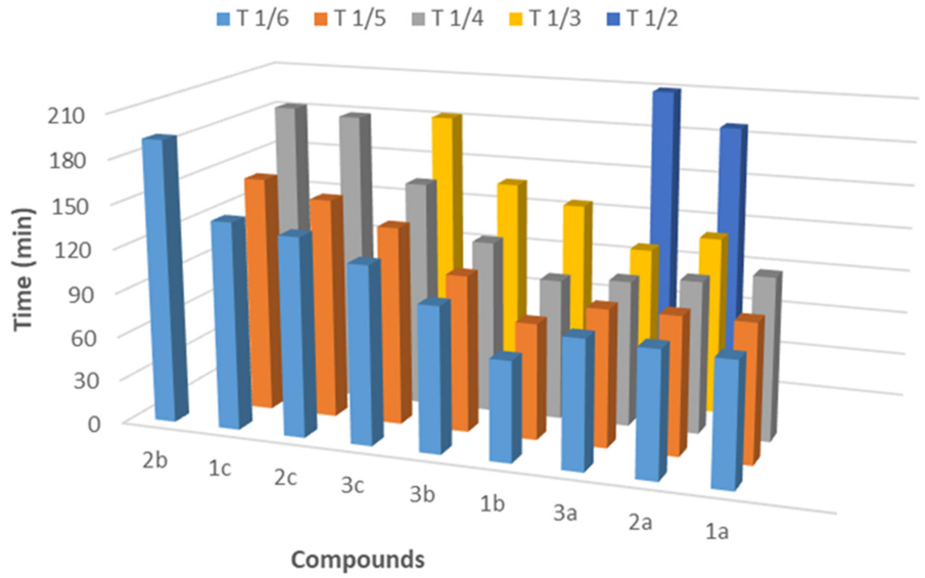

B)

		T 1/6	T 1/5	T 1/4	T 1/3	T 1/2
**2b**	**R=H ; X=O**	192,4				
**1c**	**R=OCH_3 ; X=NH**	141,3	160,0	200,3		
**2c**	**R=OCH_3 ; X=O**	135,5	149,4	196,8		
**3c**	**R=OCH_3 ; X=S**	121,2	134,6	154,0	191,3	
**3b**	**R=H ; X=S**	98,8	106,4	117,4	147,7	
**1b**	**R=H ; X=NH**	67,9	78,8	95,5	136,2	
**3a**	**R=NO_2 ; X=S**	87,3	93,2	99,2	109,3	210,0
**2a**	**R=NO_2 ; X=O**	85,5	93,5	103,7	121,1	187,8
**1a**	**R=NO_2 ; X=NH**	83,8	93,9	110,8		

Figure S6. T1/6, T1/5, T1/4, T1/3 and T1/2 values (defined as the time (min) necessary for a 20 µM CO-RM solution to produce a Mb-CO concentration equal, respectively, to 1/6, 1/5, 1/4, 1/3, and 1/2 of its initial concentration) of the analyzed compounds based on the spectrophotometric assay. The bar graph (A) represents values reported in the table (B).

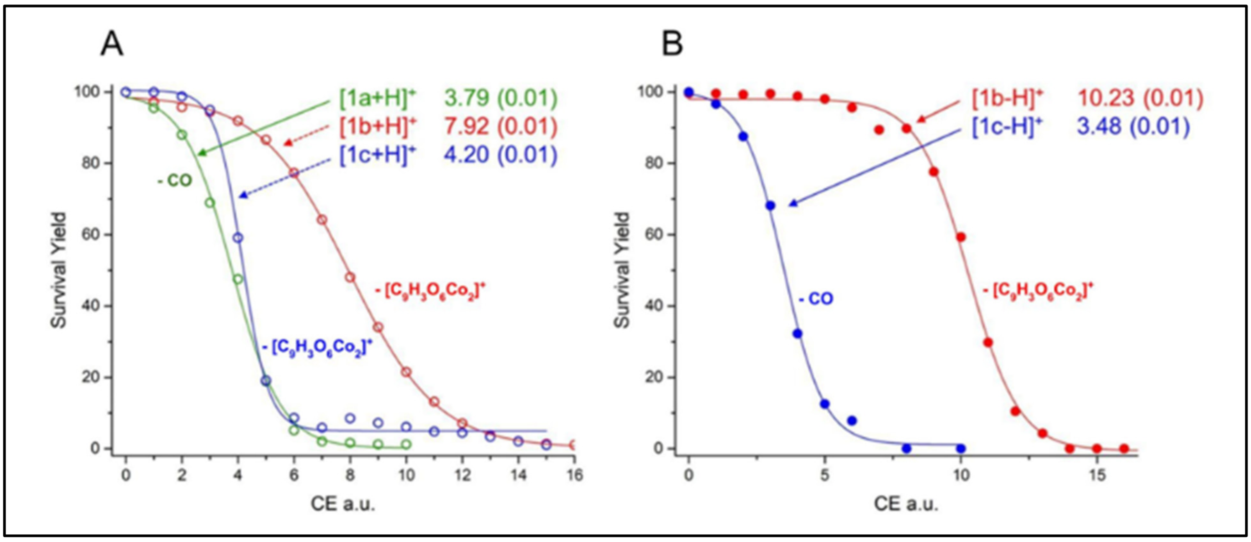

Figure S7. Survival yield curves of [**1a**+H]+, [**1b**+H]+, and [**1c**+H]+ (panel A) and [**1b**-H-]$^{+}$ and [**1c**-H-]$^{+}$ (panel B). The CID50% values (in arbitrary units, a.u.) are reported along with standard deviation in parenthesis. Note that the curves for [**1b**+H]$^{+}$, and [**1c**+H]$^{+}$ in panel A correspond to the loss of the organic portion rather than CO upon fragmentation. Curves in panel B correspond to the loss of CO. Full arrow = CO loss; dashed arrows = organic portion loss.

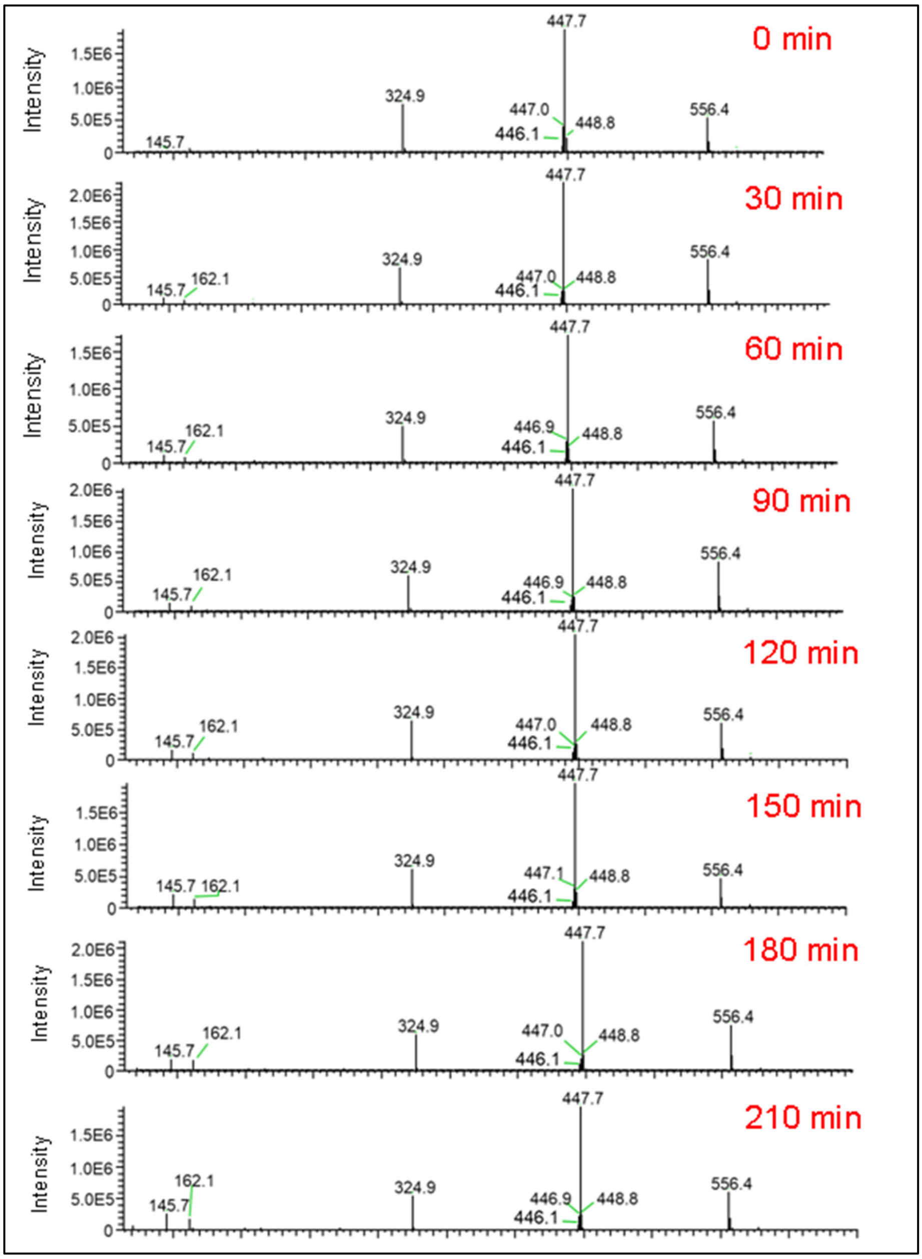

Figure S8. ESI-MS analysis of a 50 µM solution in ACN of **1c** + leucine-enkephalin + HCOOH (0.1% v/v) stored at 37 °C; the sample was analyzed at 0, 30, 60, 90, 120, 150, 180 and 210 minutes.

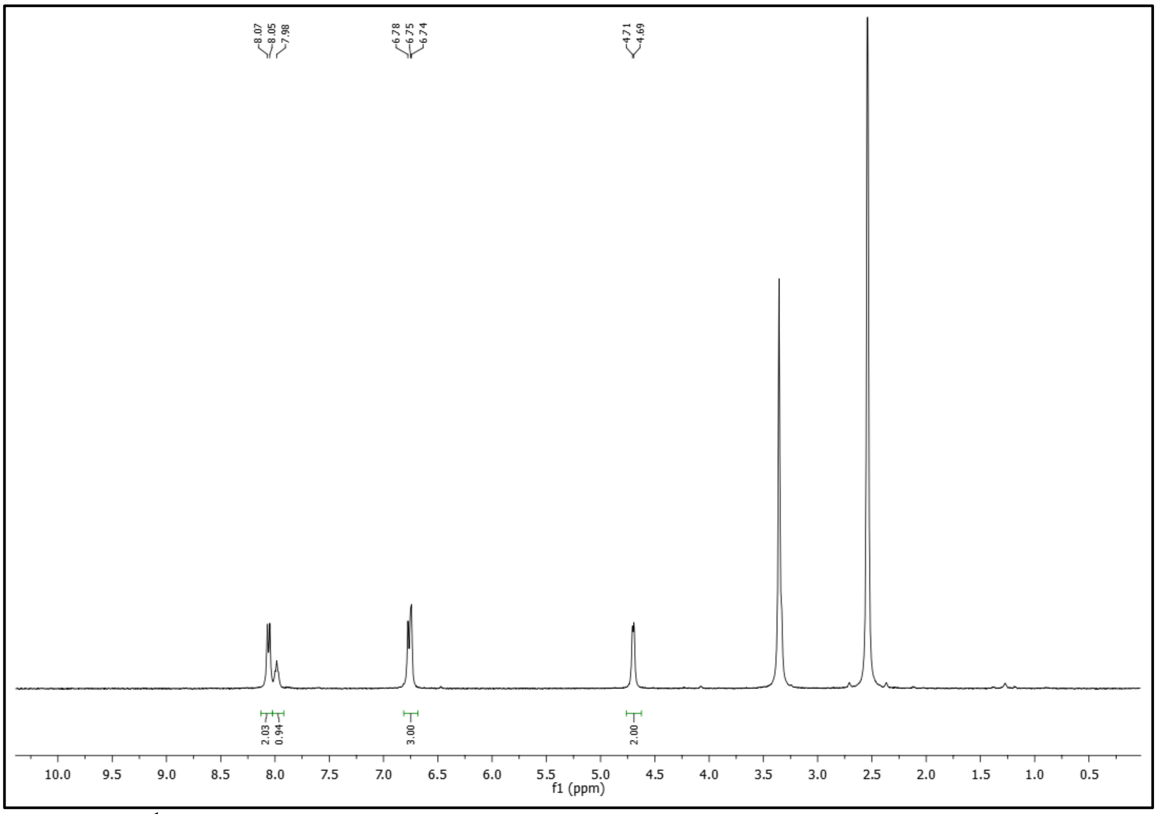

Figure S9. ^{1}H NMR of compound **1a**.

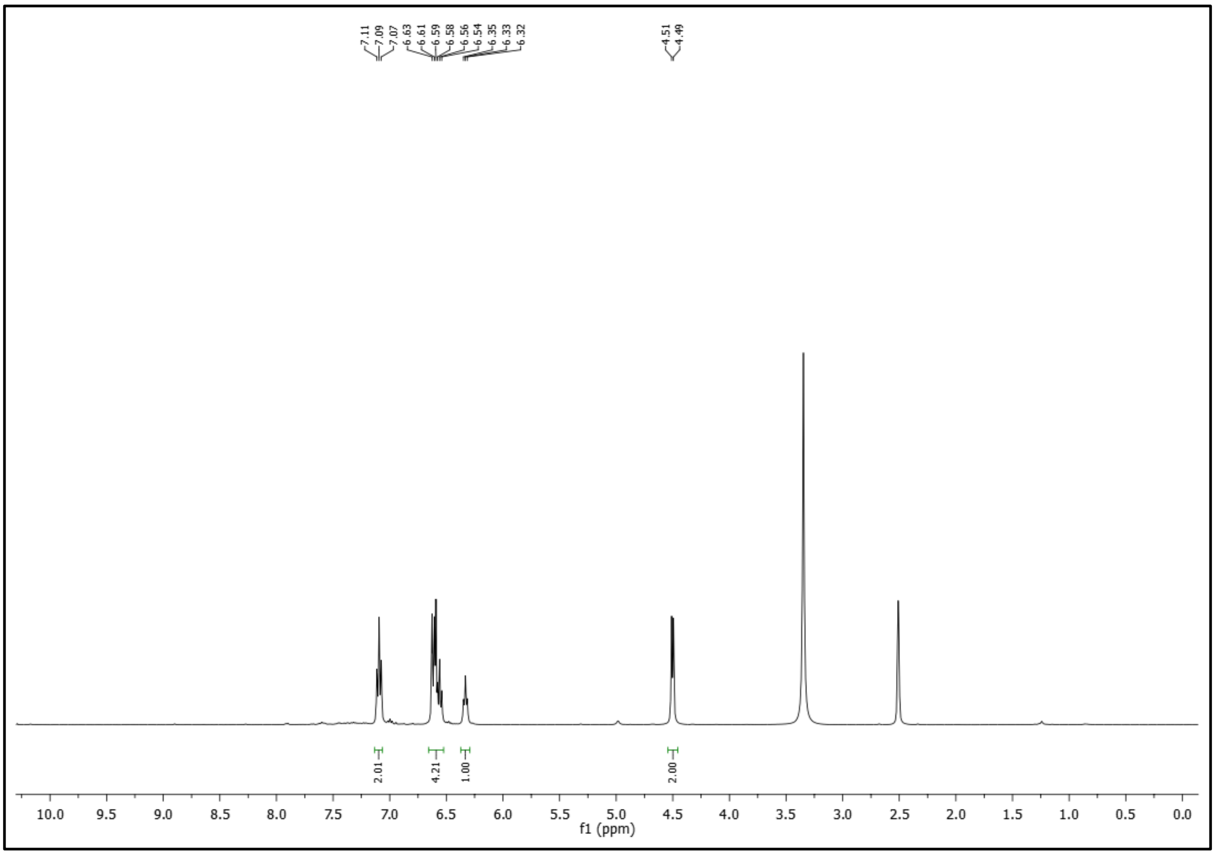

Figure S10. ^{1}H NMR of compound **1b**.

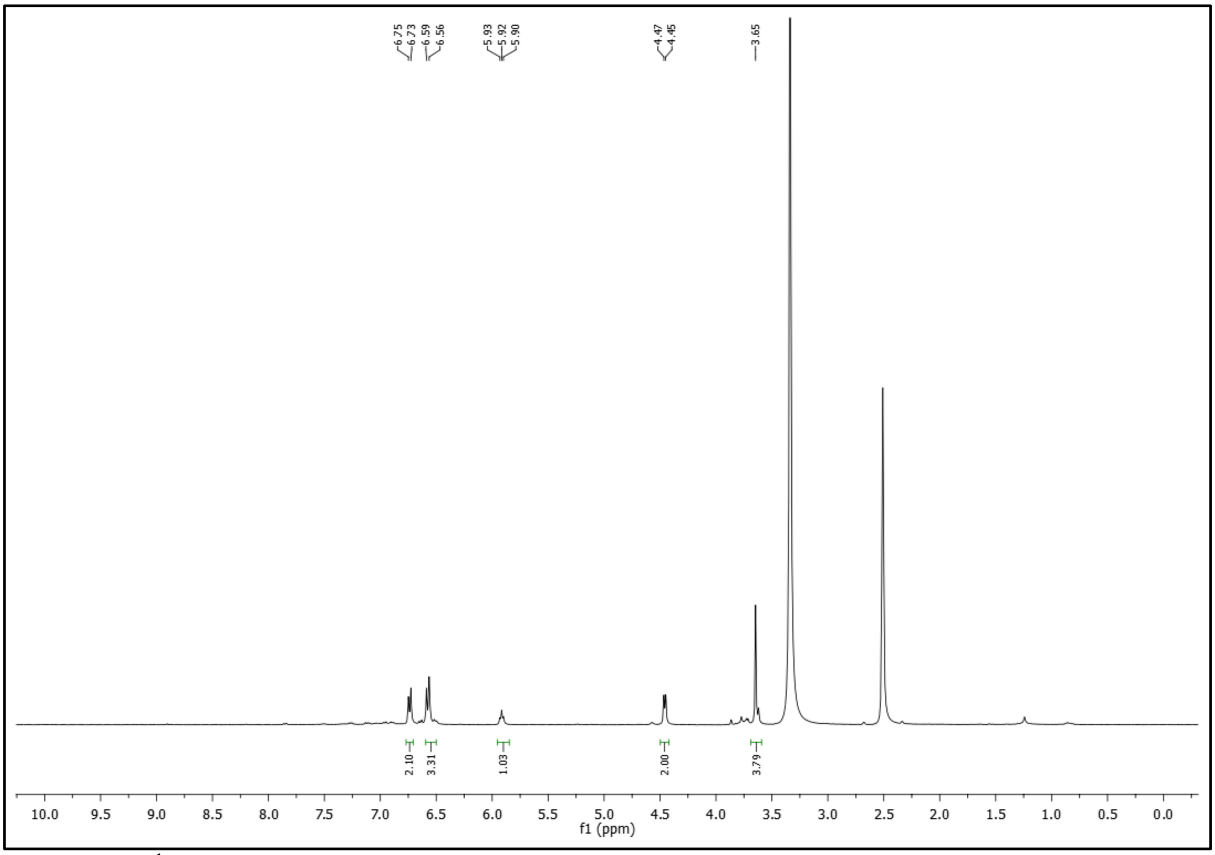

Figure S11. ^{1}H NMR of compound **1c**.

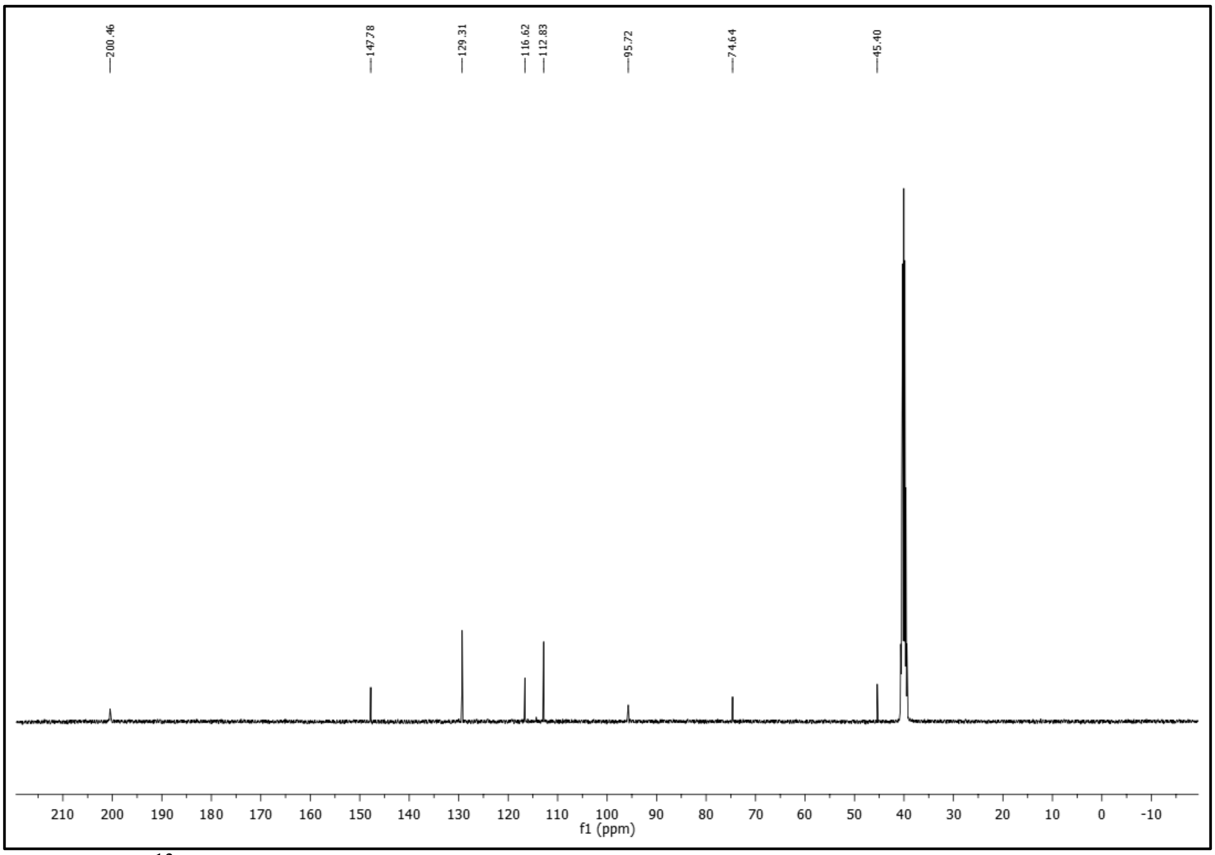

Figure S12. ^{13}C NMR of compound **1b**.

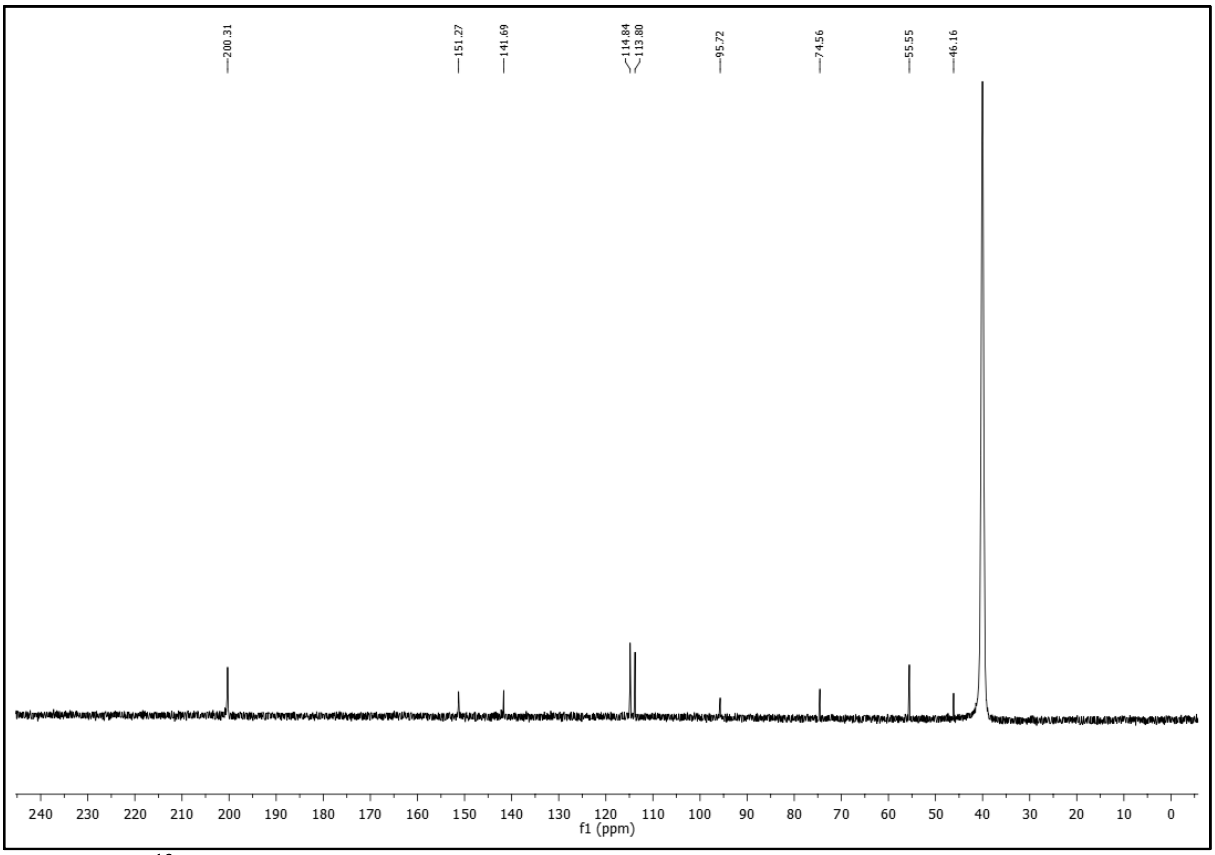

Figure S13. ^{13}C NMR of compound **1c**.

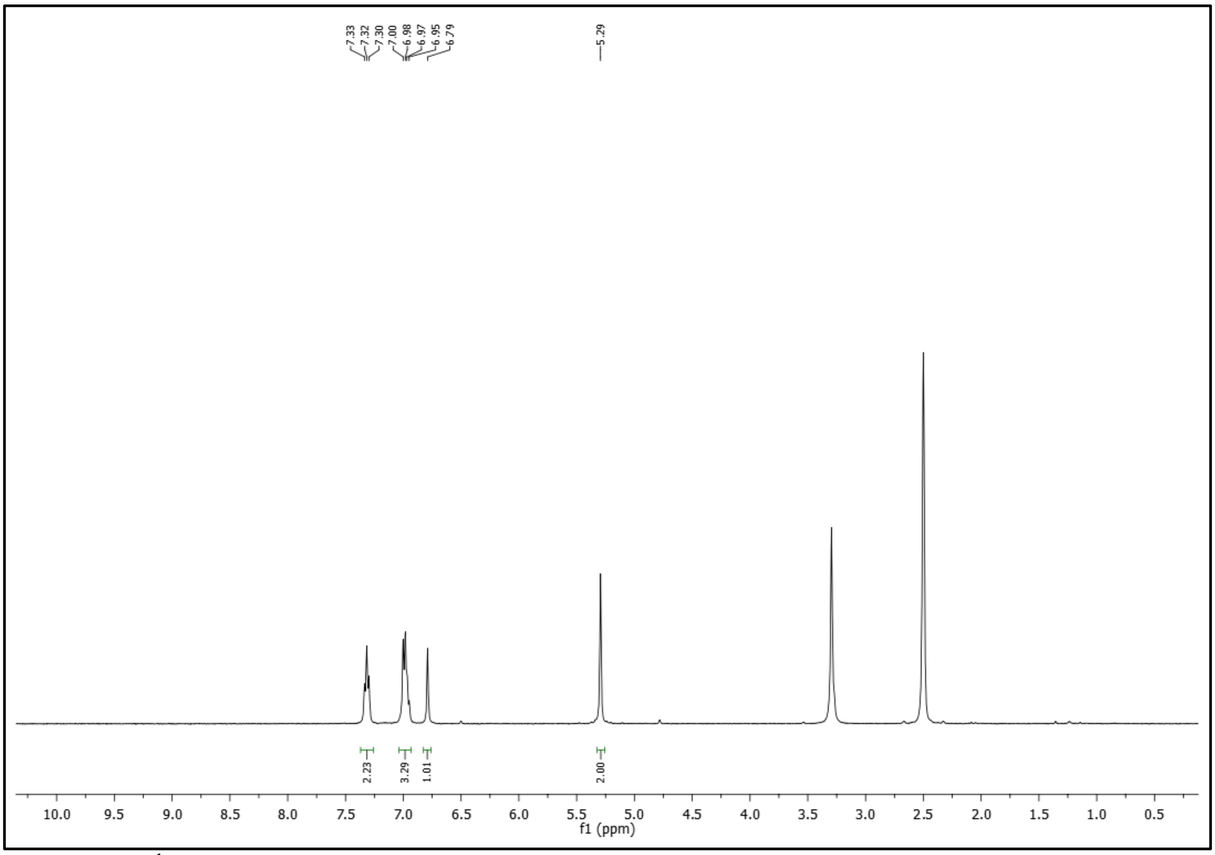

Figure S14. ^{1}H NMR of compound **2b**.

Table S1. Geometrical parameters of DCH moiety computed at B3LYP level of theory with 6-31G*/cc-pVDZ basis set.

**molecule**	**Co-Co distance (Å)**	**Co1—CO distance (Å)**			**Co2—CO distance (Å)**		
Ligand	Co-Co	**p1**	**p2**	**p3**	**p4**	**p5**	**p6**
1a	2.45	1.81	1.77	1.81	1.81	1.77	1.80
1b	2.45	1.81	1.77	1.81	1.81	1.77	1.80
1c	2.45	1.81	1.77	1.81	1.81	1.77	1.80
2a	2.45	1.81	1.77	1.81	1.81	1.77	1.81
2b	2.45	1.81	1.77	1.81	1.81	1.77	1.81
2c	2.45	1.81	1.77	1.81	1.81	1.77	1.81
3a	2.45	1.81	1.77	1.81	1.81	1.77	1.81
3b	2.45	1.81	1.77	1.81	1.81	1.77	1.81
3c	2.45	1.81	1.77	1.81	1.81	1.77	1.81

Table S2. Total stabilization energy, E^{ST} (kcal mol^{-1}), decomposed for the CO leaving groups.

Molecule	Total E^{ST} (kcal mol^{-1}) - CO leaving group					
	**p1**	**p2**	**p3**	**p4**	**p5**	**p6**
1a	17.7	20.4	16.7	16.7	20.4	18.4
1b	17.5	20.6	16.8	16.8	20.6	18.5
1c	17.5	20.6	16.9	18.0	21.5	19.2
2a	17.1	21.7	18.3	18.2	21.6	17.7
2b	17.1	21.8	18.6	18.4	21.7	17.8
2c	17.7	21.7	18.4	18.4	21.7	17.7
3a	17.0	20.6	17.2	17.7	21.4	18.1
3b	17.0	20.7	17.3	18.1	21.5	18.1
3c	16.9	20.9	17.7	18.2	21.7	17.9

Table S3. Relative Gibbs Free Energy, ΔG^{spin}, computed for each ^1A $LCo_2(CO)_5$ product with respect to the corresponding ^3A analogues.

CO position	ΔG^{spin} ($^1A - {}^3A$) - (kcal mol^{-1})								
	**1a**	**1b**	**1c**	**2a**	**2b**	**2c**	**3a**	**3b**	**3c**
p1	5.0	2.0	1.7	5.2	5.0	5.2	5.6	5.0	5.1
p2	12.3	11.9	12.0	15.5	15.7	15.8	12.8	16.0	13.3
p3	4.2	4.3	4.2	4.5	4.4	4.5	5.3	4.8	4.9
p4	4.3	4.2	4.2	4.5	4.4	4.5	2.0	4.9	4.6
p5	12.4	15.7	15.8	15.5	15.7	15.8	12.9	12.4	13.0
p6	5.3	4.8	4.7	5.2	5.0	5.2	5.4	5.3	4.6

Table S4. First Co-CO bond dissociation energy (FBDE) calculated as $\Delta H = \{H[CO] + H[LCo_2(CO)_5]\} - H[LCo_2(CO)_6]$, where $LCo_2(CO)_6$ and $LCo_2(CO)_5$ are in their ^1A and ^3A spin states, respectively. All values are reported in kcal mol^{-1}.

molecule	FBDE (kcal mol^{-1}) - CO leaving group					
	**p1**	**p2**	**p3**	**p4**	**p5**	**p6**
1a	25.1	29.8	25.1	25.1	29.8	25.1
1b	29.1	30.2	25.0	25.1	25.1	25.1
1c	29.0	30.3	25.0	25.1	25.1	25.1
2a	25.5	25.5	25.5	25.5	25.5	25.5
2b	25.4	25.4	25.4	25.4	25.4	25.4
2c	25.3	25.3	25.3	25.3	25.3	25.3
3a	24.9	29.0	24.9	28.4	28.9	24.8
3b	24.7	24.7	24.7	24.7	29.0	24.7
3c	24.6	29.1	24.6	24.7	29.5	24.7

Table S5. Gibbs free energy variation (ΔG) associated to the first carbonyl release according to $\Delta G = \{G[CO] + G[LCo_2(CO)_5]\} - G[LCo_2(CO)_6]$, where $LCo_2(CO)_6$ and $LCo_2(CO)_5$ are in their ^1A and ^3A spin states, respectively. All values are reported in kcal mol^{-1}.

CO position	ΔG^a (kcal mol^{-1})								
	**1a**	**1b**	**1c**	**2a**	**2b**	**2c**	**3a**	**3b**	**3c**
p1	18.3	21.6	21.6	18.7	18.6	18.5	17.7	17.9	17.8
p2	22.2	22.7	22.8	18.7	18.6	18.5	21.6	17.9	21.7
p3	18.3	18.2	18.2	18.7	18.6	18.5	17.7	17.9	17.8
p4	18.1	18.2	18.2	18.7	18.6	18.5	21.1	17.8	17.8
p5	22.2	18.2	18.2	18.7	18.6	18.5	21.4	21.8	21.8
p6	18.1	18.2	18.2	18.7	18.6	18.5	17.8	17.7	17.8

[a]G values were computed by applying the Werz approach (eq. 4, see "Computational details").

Table S6. Most favorable FBDE and ΔG values associated to first carbonylation dissociation calculated at the B3LYP/6-31G* (B3LYP/cc-pVDZ for the Co atom) level of theory. The complete set of FBDE and ΔG values computed for each CO position are reported in Table S4 and S5, respectively.

Molecule	FBDE (kcal mol^{-1})	ΔG (kcal mol^{-1})
**1a**	25.1	18.1
**1b**	25.0	18.2
**1c**	25.0	18.2
average	*25.0*	*18.2*
**2a**	25.5	18.7
**2b**	25.4	18.6
**2c**	25.3	18.5
average	*25.4*	*18.6*
**3a**	24.8	17.7
**3b**	24.7	17.8
**3c**	24.6	17.8
average	*24.7*	*17.8*

Table S7. First Co-CO bond dissociation energy (FBDE) computing as ΔH= {H[CO] + H[$LCo_2(CO)_5$]} - H[$LCo_2(CO)_6$], where $LCo_2(CO)_6$ and $LCo_2(CO)_5$ are both in their ^{1}A spin state. All values are reported in kcal mol^{-1}. The average value of the most stable FBDEs is 28.0 ± 0.2 kcal mol^{-1}.

molecule	FBDE (kcal mol^{-1}) - CO leaving group					
	p1	p2	p3	p4	p5	p6
1a	28.8	29.4	28.0	27.9	39.4	28.8
1b	28.6	40.1	28.0	27.8	39.6	29.8
1c	28.5	40.2	28.0	27.7	39.7	28.8
2a	29.2	39.7	28.3	28.3	39.7	29.2
2b	29.1	39.9	28.1	28.1	39.9	29.1
2c	29.1	40.0	28.1	28.1	40.0	29.1
3a	28.3	39.0	27.9	27.9	39.0	28.3
3b	28.1	39.2	27.7	27.7	39.2	28.1
3c	28.4	39.7	27.9	27.9	39.7	28.4

Table S8. Complete set of analyzed and detected CO-RMs by means of ESI-MS/MS. CID fragments, used to confirm the sampled ion identity, are also reported. The identity of **3a** and **3b** was only confirmed through detection of the $[C_{12}H_8Co_2O_3S]^+$ and $[C_{12}H_9NCoO_5S]^+$ species respectively.

	Detected Ion	Exp. *m/z* [a]	Theor. *m/z* [b]	CID fragment
**1a**	$[M+Na^+]^+$	484.8	484.88369	456.8, 428.8, 400.8
	$[M+K^+]^+$	500.8	500.85762	472.8, 444.8, 416.7
**1b**	$[M-H^-]^+$	415.9	415.90101	387.8, 359.6, 331.6, 303.7
	$[M+H^+]^+$	417.9	417.91666	324.9, 296.9
**1c**	$[M-H^-]^+$	445.9	445.88782	417.7, 389.7, 361.7
	$[M+H^+]^+$	447.9	447.90341	324.9, 296.9
**2a**	$[M+Na^+]^+$	485.9	485.86770	457.8
**2b**	-	-	417.8934	-
**2c**	-	-	448.91124	-
**3a**	$[C_{12}H_9NCoO_5S]^+$	338.0	337.95279	-
	$[M+H^+]^+$	462.9	462.90174	434.9, 406.9, 378.9
**3b**	$[C_{12}H_8Co_2O_3S]^+$	349.9	349.88527	321.9, 293.9
**3c**	$[M+K^+]^+$	502.8	502.84434	474.8, 446.8,418.8

[a] experimental *m*/*z*; [b] theoretical *m*/*z*.

Table S9. First order rate constants, *k*, and first carbonyl dissociation energy, ΔE^{exp}, computed by using the experimental data of CO release assay. All the released CO molecules are assumed to be captured by Mb.

molecule	ΔE^{exp} (kcal mol^{-1}) [a]	*k* (s^{-1}) [b]
1a	24.7	2.62 x 10^{-5}
1b	24.3	4.68 x 10^{-5}
1c	25.0	1.40 x 10^{-5}
average	*24.7*	
2a	24.7	2.29 x 10^{-5}
2b	25.1	1.34 x 10^{-5}
2c	25.1	1.22 x 10^{-5}
average	*25.0*	
3a	24.6	1.85 x 10^{-5}
3b	24.9	2.93 x 10^{-5}
3c	25.4	2.05 x 10^{-5}
average	*25.0*	

[a] ΔE^{exp} were computed by including the first order rate constant, *k*, in the Arrhenius equation ($k = A\exp\{\Delta E^{exp}/RT\}$) where A is 6.25 x 10^{12} molecule/s (C. J. Cramer, Essentials of Computational Chemistry: Theories and Models, John Wiley & Sons, New York, 2004, pp. 519–528).

[b] Rate constants were estimated as the slope of the line obtained by plotting the concentration of the DCH derivatives (ln[C]) as a function of time (s). Only the first 60' of Mb assay data were considered to avoid the interference of possible side reactions.
